# ZFP36L2 regulates myocardial ischemia/reperfusion injury and attenuates mitochondrial fusion and fission by LncRNA PVT1

**DOI:** 10.1038/s41419-021-03876-5

**Published:** 2021-06-15

**Authors:** Fang Wu, Weifeng Huang, Qin Tan, Yong Guo, Yongmei Cao, Jiawei Shang, Feng Ping, Wei Wang, Yingchuan Li

**Affiliations:** Department of Critical Care Medicine, Shanghai Jiaotong University Affiliated Sixth People’s Hospital, 200233 Shanghai, China

**Keywords:** Heart failure, Heart failure

## Abstract

Among several leading cardiovascular disorders, ischemia–reperfusion (I/R) injury causes severe manifestations including acute heart failure and systemic dysfunction. Recently, there has been increasing evidence suggesting that alterations in mitochondrial morphology and dysfunction also play an important role in the prognosis of cardiac disorders. Long non-coding RNAs (lncRNAs) form major regulatory networks altering gene transcription and translation. While the role of lncRNAs has been extensively studied in cancer and tumor biology, their implications on mitochondrial morphology and functions remain to be elucidated. In this study, the functional roles of Zinc finger protein 36-like 2 (ZFP36L2) and lncRNA PVT1 were determined in cardiomyocytes under hypoxia/reoxygenation (H/R) injury in vitro and myocardial I/R injury in vivo. Western blot and qRT-PCR analysis were used to assess the levels of ZFP36L2, mitochondrial fission and fusion markers in the myocardial tissues and cardiomyocytes. Cardiac function was determined by immunohistochemistry, H&E staining, and echocardiogram. Ultrastructural analysis of mitochondrial fission was performed using transmission electron microscopy. The mechanistic model consisting of PVT1 with ZFP36L2 and microRNA miR-21-5p with E3 ubiquitin ligase MARCH5 was assessed by subcellular fraction, RNA pull down, FISH, and luciferase reporter assays. These results identified a novel regulatory axis involving PVT1, miR-21-5p, and MARCH5 that alters mitochondrial morphology and function during myocardial I/R injury. Using an in vivo I/R injury mouse model and in vitro cardiomyocytes H/R model, we demonstrated that ZFP36L2 directly associates with PVT1 and alters mitochondrial fission and fusion. PVT1 also interactes with miR-21-5p and suppresses its expression and activity. Furthermore, we identified MARCH5 as a modifier of miR-21-5p, and its effect on mitochondrial fission and fusion are directly proportional to PVT1 expression during H/R injury. Our findings show that manipulation of PVT1-miR-21-5p-MARCH5-mediated mitochondrial fission and fusion via ZFP36L2 may be a novel therapeutic approach to regulate myocardial I/R injury.

## Introduction

Hypoperfusion of the heart for short periods of time in response to sepsis, transplantation, or other syndromes is known as ischemia. Subsequent reperfusion or restoration of blood flow in some conditions causes injuries to ischemic tissues resulting in ischemia–reperfusion (I/R) injury. Prolonged I/R injury can lead to myocardial infarction and acute coronary syndrome [1]. Prolonged hypoxic conditions lead to anaerobic respiration and dysregulation of the electron transport chain in mitochondria thus resulting in lower levels of ATP, release of stress factors and generation of anti-oxidative reactive oxygen species (ROS) [2] thereby triggering cellular dysfunction, DNA damage, and apoptosis [3]. In the process of apoptosis, mitochondria are fragmented (mitoptosis), which is regulated by several proteins including dynamin related protein 1 (Drp1) and fission 1 (Fis1) [4].

Plasmacytoma variant translocation 1 (PVT1) belongs to the class of long non-coding RNAs (lncRNAs) that are classically differentiated from other non-coding microRNAs (miRNAs) based on its size, where lncRNAs are typically greater than 200 nucleotides in length whereas miRNAs are ~22 nucleotides in length. PVT1 has been implicated in Burkett’s lymphoma and its oncogenic functions has been studied for decades [4–7]. Aberrant expression of PVT1 and its ability to regulate several miRNAs are hallmarks of cancer invasion and progression [8, 9]. In the context of cellular degradation, lncRNAs have been extensively characterized for its role in autophagy through interaction with miRNAs. For example, miR-30a inhibition promoted autophagy protecting neuronal fibers from I/R injury [10]. However, the role of lncRNAs in the regulation of mitochondrial fission/fusion processes during cell death has not been explored so far. In a study by Alessio et al. microarray analysis in single cell muscle fibers identified PVT1 as a regulator of mitochondrial respiration, fission/fusion, and mitophagy/autophagy [11].

Zinc finger protein 36-like 2 (ZFP36L2) is part of a family of proteins that contain tandem zinc finger domains that can associate with adenine–uridine rich elements present most often in the 3′-untranslated region (UTR) of mRNAs thereby interfering with posttranscriptional modifications and affecting protein translation [12]. It causes cell cycle arrest, especially during embryonic development and has recently been shown to alter immune functions in T cells [13–15]. ZFP36L2 also regulates adipogenesis and development of B cells. ZFP36L2 mediates anti-inflammatory effects on lipopolysaccharide (LPS)-induced acute lung injury, and also protects hepatic IR injury, which is driven by the generation of ROS [16, 17]. So far, regulatory roles of ZFP36L2 in cardiomyocytes and in the context of I/R injury have not been described.

On the other hand, miRNAs and their role in altering mitochondrial morphology and functions in cardiomyocytes have been broadly studied. Mitochondrial miRNAs are targeted towards regulating pathways related to cell apoptosis, proliferation and differentiation [18]. In the context of processes related to mitochondrial morphogenesis, miR-200a-3p has been shown to promote mitochondrial elongation by targeting mitochondrial fission factor (Mff) [19]. Using an antitumor drug, doxorubicin-induced cardiomyopathy can be counteracted using miR-532-3p, which targets mitochondrial fission and fusion processes in cardiomyocytes [20]. In I/R injury, several miRNAs including miR-140 and miR-15 have been shown to alter mitochondrial fission/fusion and apoptosis [21–25]. Mitochondrial RING-finger E3 ubiquitin-protein ligase MARCH5 (or MITOL) regulates mitochondrial morphogenesis by direct ubiquitination and degradation of fission proteins Fis1, Mfn1, and Mfn2 [26, 27]. Under hypoxia, MARCH5 ubiquitinates mitochondrial receptor FUNDC to prevent hypoxia-induced mitophagy [28, 29].

In this study, we investigated that ZFP36L2 directly binds to PVT1 and altered mitochondrial fission and fusion, and PVT1 interacts with miR-21-5p and suppresses its expression. MARCH5 was identified as the target of miR-21-5p. In conclusion, ZFP36L2-PVT1-miR-21-5p-MARCH5 axis played an important role in mitochondrial fission and fusion processes during I/R injury.

## Materials and methods

### Culture and treatment of cardiomyocytes

Cardiomyocytes were isolated from male mice (1–2 days old) purchased from Shanghai Jiaotong University Affiliated Sixth People’s Hospital. Briefly, hearts were dissected, washed and cut into small pieces in HEPES-buffered saline (HBS). The tissues were then digested with pancreatin (1.2 mg/ml) and collagenase (0.14 mg/ml) at 37 °C in HBS. The resulting supernatants were collected and centrifuged at 200 × *g* for 5 min. The cells were collected and incubated in MDEM/F-12 medium (GIBCO) containing 5% heat-inactivated horse serum, 0.1 mM ascorbate, insulin-transferring-sodium selenite media supplement (Sigma), 100 U/ml penicillin, 100 μg/ml streptomycin, and 0.1 mM bromodeoxyuridine at 37 °C for 24 h. After 24 h of culture, non-adherent cells and debris were aspirated. When the cells were reached 80%, the cells were passaged and changed the medium. The in vitro experiments were created within five passages, and all experiments were completed within ten passages. Before the further in vitro experiments, the cells were validated by STR profiling from Procell Life Science & Technology (Wuhan, China).

Cells were cultured in vitro in Dulbecco’s Modified Eagle’s Medium supplemented with 5% heat-inactivated horse serum and antimycotic cocktail (Thermo Fisher Scientific, Waltham, MA, USA), and plated on laminin-coated (10 μg/mL) culture dishes. Cells were transduced with either non-targeting shRNA (NC), or shRNA against ZFP36L2, PVT1, or MARCH5 for 48 h. Cells were transfected with a PVT1 overexpression construct using Lipofectamine 2000 (Invitrogen, Carlsbad, CA, USA) according to the manufacturer’s instructions. For the hypoxia/reoxygenation (H/R) based injury model, cardiomyocytes were stimulated with palmitate (100 μM) for 2 h followed by induction of hypoxia (1% O_2_) for 4 h and reoxygenation for 1 h. Cells were then stained for further mitochondrial analyses or harvested to evaluate mRNA and protein expression.

### Myocardial I/R injury model

All animal experiments were conducted in accordance with the requirements and principles of the Animal Care and Use Committee of Shanghai Jiaotong University Affiliated Sixth People’s Hospital and performed according to established guidelines. 8-week-old C57BL/6 wild-type male mice were anesthetized using isoflurane to perform thoracotomy. The mice were grouped randomly (six mice per group). Before the myocardial I/R injury, we collected the blood of the mice, the mice were excluded if the cardiac dysfunction (LDH and CK-MB levels) was significantly elevated. To assess myocardial I/R injury, mice were subjected to 45 min myocardial ischemia followed by 4 h reperfusion. Sham-operated group underwent the same procedure except that the snare was left untied. After reperfusion, Evans blue dye (1 ml of a 2% solution; Sigma-Aldrich) was injected through jugular vein to delineate the ischemic area at risk. The mice were euthanized by cervical dislocation and the heart was rapidly excised and sectioned. The heart slices were incubated in 1.0% 2, 3, 5-triphenyltetrazolium chloride (TTC; Sigma-Aldrich) for 15 min at 37 °C to differentiate live (red) from dead or infarcted myocardium (white). After washing in ice-cold sterile saline, the slices were fixed in 10% formaldehyde, weighed and photographed from both sides. The infarct area (INF) and the risk zone were assessed using computer-assisted planimetry by a histologist blinded to treatment conditions. The INF/LV ratio (%) LVIDd (mm) and the infarct size (defined as % of risk zone) were calculated.

To observe the influence of ZFP36L2, PVT1, miR-21-5p, and MARCH5 on the myocardial I/R injury, the mice were randomly divided in to four groups: (1) sham, I/R, I/R + shRNA NC, I/R + ZFP36L2 shRNA, (2) sham, I/R, I/R + sh-NC, I/R + sh-PVT1, and (3) sham, I/R, I/R + sh-NC, I/R + sh-MARCH5. For intracoronary delivery of adenoviruses, mice were anesthetized and intubated, and ventilated with a HX-300S animal ventilator. The heart was exposed through a small left anterior thoracotomy. Adenoviruses carrying (1) shRNA-NC (200:1 MOI), ZFP36L2 shRNA (200:1 MOI), (2) sh-NC (250:1 MOI), sh-PVT1 (250:1 MOI), and (3) sh-NC (250:1 MOI), sh-MARCH5 (250:1 MOI) were injected with a catheter from the LV apex into the aortic root while the aorta and pulmonary arteries were cross-clamped for 20 s. The chest was closed after removal of air and blood and the mice were allowed to recover. Five days after the injection of adenoviruses, the mice were subjected to I/R treatment.

### Quantitative reverse transcription-PCR

Total RNA was extracted from heart tissues or cells using a RNEase kit (Qiagen, Hilden, Germany) according to the manufacturer’s protocol. A total of 500 ng of RNA was used for subsequent cDNA preparation and quantitative PCR using SYBR-Green (Thermo Fisher Scientific). The results were normalized to glyceraldehyde 3-phosphate dehydrogenase (GAPDH). The primer sequences used for amplification of DRP1, Fis1, Mff, Mfn1, Mfn2, ZFP36L2, PVT1, miR-21-5p, MARCH5, and GAPDH are listed in Supplementary Table S1.

### Western blot analysis

Protein lysates from cells or tissues were quantified using a BioDrop μLITE analyzer (BioDrop, Cambridge, UK). A total of 50 μg of protein was loaded onto 10% SDS gels and blotted onto nitrocellulose membranes. The membranes were incubated in primary antibodies against Drp1 (1:1000, ab184247, Abcam, UK), Fis1 (1:500, ab189846, Abcam, UK), Mff (1:1000, 86668S, Cell signaling Technology, USA), Mfn1 (1:2000, ab107129, Abcam, UK), Mfn2 (1:1000, ab124773, Abcam, UK), ZFP36L2 (1:500, 12306-1-AP, Proteintech, China), MARCH5(1:1000, 19168S, Cell signaling Technology, USA), and GAPDH (1:500, ab8245, Abcam, UK) overnight at 4 °C. After washing in phosphate-buffered saline-Tris (PBS-T), the membranes were incubated in fluorescent secondary antibodies for 1 h and visualized using an Odyssey Imager (LiCor, Lincoln, NE, USA). Image analysis and quantification were performed using Image J software (National Institutes of Health, Bethesda, MD, USA).

### Histology

Heart sections were analyzed using hematoxylin and eosin (H&E) staining and immunohistochemistry (IHC). Samples were prepared for histology by infusing 4% paraformaldehyde into the heart. 5 μm microsections were then subsequently stained with H&E or Masson’s trichome to assess infiltration and fibrosis. For IHC, tissue sections were stained using primary antibodies against ZFP36L2 or MARCH5 overnight at 4 °C and imaged using a light microscope.

### Subcellular fractionation

Cells were washed with fresh PBS and resuspended in fractionation buffer [20 mmol HEPES (pH 7.5), 10 mmol KCl, 1.5 mmol MgCl_2_, 1 mmol EGTA, 1 mmol EDTA, 1 mmol DTT, and 0.1 mmol phenylmethanesulfonyl fluoride], 250 mmol sucrose, and 20 mmol protease inhibitor cocktail (Sigma-Aldrich, St. Louis, MO, USA). Cell suspension was homogenized using a Dounce homogenizer and centrifuged at 750 × *g* for 5 min. The nuclei were pelleted, leaving the cytosolic and ER fraction in the supernatant. The supernatant was subsequently centrifuged at 10,000 × *g* for 15 min to pellet the mitochondrial fraction. A third centrifugation step of the supernatant yielded the cytosolic fraction. The fractions were analyzed for protein expression using western blot.

### Biotinylated miRNA pull-down assay

RNA pull-down assay was performed as described previously [30]. Briefly, cells were transfected with biotinylated miR-21-5p, miR-21-5p-mut, or ZFP36L2, and harvested 48 h later for lysis. Cell lysates were incubated with streptavidin magnetic beads for 3 h at 4 °C. The beads were thoroughly washed with a low salt buffer (0.1% SDS, 1% Triton X-100, 2 mM EDTA, 20 mM Tris-HCl, pH 8.0, and 150 mM NaCl) followed by a high salt buffer ((0.1% SDS, 1% Triton X-100, 2 mM EDTA, 20 mM Tris-HCl, pH 8.0, and 500 mM NaCl). All bound RNA molecules were analyzed by northern blotting.

### DNA pull-down assay

A complementary DNA probe (biotinylated) to PVT1 was synthesized (Sigma-Aldrich) and incubated with streptavidin-coated magnetic beads in binding buffer. DNA bound beads were then used to pull-down RNA from lysates prepared from cardiomyocytes. RNA bound to the probe was eluted and analyzed using northern blotting.

### Luciferase assay

*pvt1* and *march5* wild-type and mutant sequences were expressed using a pGL3 vector (Promega, Madison, WI, USA) encoding the firefly luciferase gene. Cardiomyocytes were co-transfected with the luciferase constructs using Lipofectamine 2000 (Invitrogen), and cells were harvested 48 h post transfection for analysis using the Dual luciferase Reporter Assay kit (Promega) according to the manufacturer’s instructions. 30 μL protein samples were analyzed in a luminometer. Firefly luciferase activities were normalized to Renilla luciferase activity.

### Northern blot analysis

Samples were subjected to polyacrylamide-urea gel electrophoresis, blotted onto positively charged nylon membranes, and cross-linked using UV irradiation. Membranes were hybridized using 100 pmol 30-digoxigenin (DIG)-labeled probes against ZFP36L2, PVT1, or miR-21-5p overnight at 4 °C and detected using a DIG luminescent detection kit (MyLab) according to the manufacturer’s instructions.

### Immunofluorescence assay

Cells were seeded onto poly-L-lysine coated coverslips, fixed with 4% paraformaldehyde, permeabilized with 0.1% Triton X-100, and blocked using standard 5% bovine serum albumin in PBS (blocking solution) and incubated with primary antibody against Drp1 (1:1000, Abcam, UK) for 1 h at room temperature. After washing three times using PBS, the coverslips were incubated with Alexa Fluor 488-labeled goat anti-rabbit secondary antibody (1:1000, Abcam, UK) for 1 h at room temperature. The antibody was washed away three times with PBS and the samples were mounted for visualization and imaged using confocal microscopy.

### Fluorescence in situ hybridization (FISH)

End-labeled 6-carboxyfluorescein (FAM) probes were synthesized for PVT1 and ZFP36L2 (Invitrogen). Cells were seeded on coverslips and fixed using 10% neutral-buffered formalin and resuspended in hybridization buffer [0.7 M NaCl, 0.1 M Tris (pH 8.0), 0.1% SDS, and 10 mM EDTA] containing the probes. Samples were heated at 55 °C for 30 min and unbound probes were washed using probe-free hybridization buffer. Cells were counterstained and mounted using mounting medium containing 4ʹ, 6-diamidino-2-phenylindole (DAPI). Coverslips were analyzed by fluorescence microscopy (Carl Zeiss, Jena, Germany).

### Mitochondrial staining

Cells were seeded onto poly-L-lysine coated coverslips, stained with MitoTracker Red CMXRos (0.02 μM; Molecular Probes, Eugene, OR, USA), and analyzed using a confocal microscope (LSM 510 META; Zeiss, Oberkochen, Germany). Total cells with fragmented mitochondria were represented as a percentage by counting at least 300 cells per treatment group from six different fields of view.

### Measurement of mitochondrial function

A mitochondrial membrane potential assay kit with JC-1 (Beyotime, China) was used to detect changes in the mitochondrial membrane potential (△Ψm). The experiment was performed according to the manufacturer’s protocol. Mitochondrial Adenosine Triphosphate (ATP) levels and mitochondrial ROS were measured using luciferin-luciferase assay kit (Beyotime, China) and ROS detection kit (Beyotime, China), respectively, according to manufacturer’s instructions. Mitochondrial oxygen consumption rate (OCR) was tested by sequentially adding 2 μM oligomycin (an ATP synthase blocker), 1 μM carbonyl cyanide 4-(trifluoromethoxy) phenylhydrazone (FCCP, the mitochondrial uncoupler), 1 μM antimycin A, and 1 μM rotenone (R, inhibitors of complex I and III). The XFe Wave software (Seahorse Biosciences) was used to quantify results.

### Electron microscopy

Ultrastructural analysis of mitochondrial fission was performed using transmission electron microscopy (TEM) as described previously [31]. Tomograms from sections were obtained using a JEM-1230 transmission electron microscope (JEOL, Tokyo, Japan) and analyzed using Image-Pro Plus software (Media Cybernetics, Rockville, MD, USA). More than 1200 mitochondrion were analyzed to determine the mitochondria sizes. Structures <0.6 mm^2^ were classified as mitochondria undergoing fission.

### Statistical analysis

All data are expressed as the mean (±SD) of at least three independent experiments, and statistical significance was calculated using two tailed Student’s *t* test to compare two groups. Mann–Whitney test was used to analyze the data with non-Gaussian distribution. The Gaussian distribution data was determined by the *F*-test, when significantly different. And one-way ANOVA analysis of variance was used to compare multiple groups. A value of *P* < 0.5 was considered statistically significant.

## Results

### ZFP36L2 knockdown inhibites myocardial ischemia/reperfusion (I/R) injury and attenuates mitochondrial fission in vivo

**Fig. 1:**
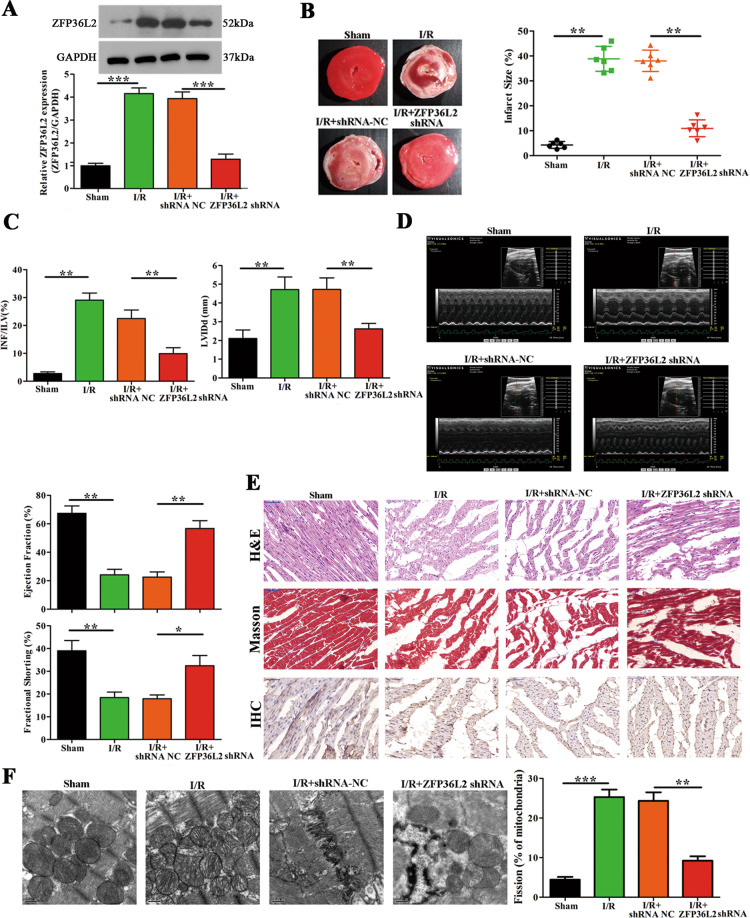
ZFP36L2 knockdown inhibites myocardial I/R injury and attenuates mitochondrial fusion and fission in vivo. **A** A myocardial I/R injury model was established by inducing ischemia for 45 min following 4 h of reperfusion (*n* = 6 per group). ZFP36L2 shRNA and shRNA-NC were intraperitoneally administered 24 h before induction of I/R injury. RNA was isolated from cardiomyocytes and mRNA and protein levels of ZFP36L2 were determined by qRT-PCR and western blot (*n* = 6). **B** The left panel shows representative images of tissue samples. The right panel indicates infarction size (%) (*n* = 6). **C** Quantification of the extent of injury measured by infarction area/left ventricle (INF/LV) and diastolic diameter of the left ventricle (LVIDd) (*n* = 6). **D** Representative echocardiograms at 4 h post I/R injury. The percentage of ejection fraction (EF) and fractional shorting (FS) were shown (*n* = 6). **E** Representative images of H&E and Masson’s trichrome staining and immunohistochemistry staining for ZFP36L2 in LV sections (Scale bar = 50 μm) (*n* = 6). **F** Representative TEM images of heart tissues shown in the left panel and the corresponding quantification of fragmented mitochondria shown in the right panel (Scale bar = 0.5 μm) (*n* = 6). *P < 0.05; **P < 0.01; ***P < 0.001.

Regulatory functions are imposed by RNA binding proteins in response to cellular stress, which alters transcription and translation [32]. In an in vivo mouse model, upon induction of ischemia followed by reperfusion, a 3-fold increase in ZFP36L2 protein expression was observed. This increase was reduced upon treatment with a shRNA targeted against ZFP36L2 (Fig. 1A). Reducing the levels of ZFP36L2 significantly lowered infarct size, infarct area/left ventricle (INF/LV), and improved the functioning of the left ventricle as measured by the left ventricular internal diastolic diameter (LVIDd) (Fig. 1B, C). Conversely, the effect of ZFP36L2 overexpression resulted in a significant increase in the infarct size (Supplementary Fig. S1A). An echocardiogram showed reduced capacity of ejection fraction and fractional shortening upon I/R induction, which was significantly improved upon knockdown of ZFP36L2 (Fig. 1D). Analyses of cardiomyocytes by H&E and Masson’s staining revealed that I/R injury caused discontinuous tissue architecture due to extensive apoptosis, but knockdown of ZFP36L2 alleviated this effect (Fig. 1E). Ultrastructural analyses using TEM showed that mitochondrial fission was significantly increased upon I/R injury, as also indicated by increased expression of fission protein: Drp1, Fis1, and Mff, and a decrease in fusion proteins: Mfn1 and Mfn2. This effect was dependent on ZFP36L2 (Fig. 1F and Supplementary Fig. S1B). Together, these results identify ZFP36L2 as a key regulator of myocardial injury during I/R.

### ZFP36L2 knockdown attenuates cardiomyocytes hypoxia/reoxygenation (H/R) injury and mitochondrial function in vitro

**Fig. 2:**
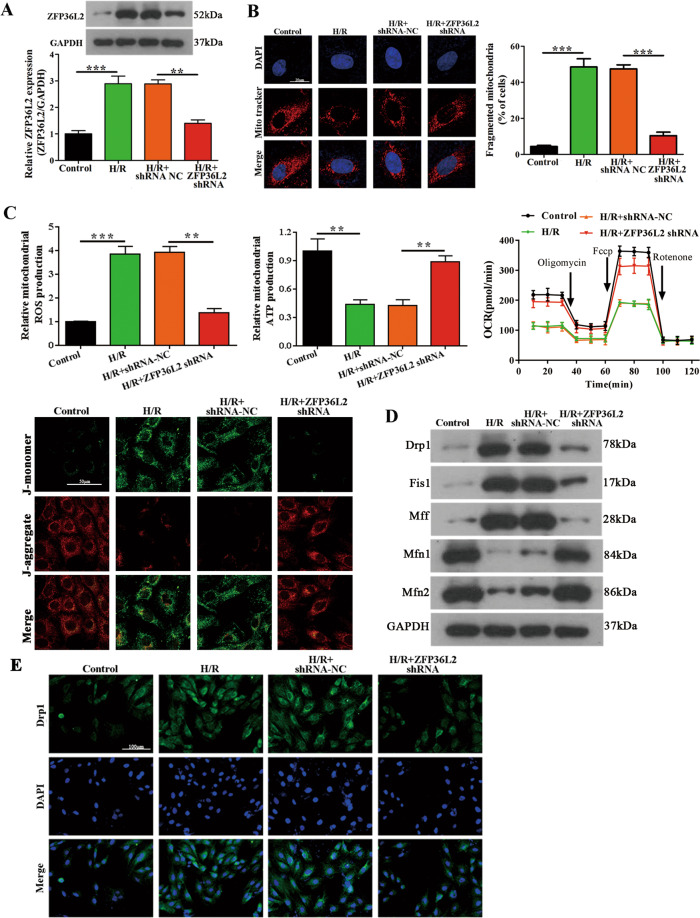
ZFP36L2 knockdown suppresses hypoxia/reoxygenation (H/R) injury and alleviates mitochondrial fusion and fission in vitro. **A** A H/R injury model was established by inducing hypoxia for 4 h followed by reoxygenation for 1 h. The mRNA and protein expression of ZFP36L2 in cardiomyocytes were determined by qRT-PCR and western blot, respectively (*n* = 3). **B** Representative micrographs of cardiomyocytes after H/R injury expressing normal control shRNA or shRNA against ZFP36L2 stained with MitoTracker Red (mitochondria) and 4′, 6-diamidino-2-phenylindole (DAPI) (nucleus). Quantification of cells with fragmented mitochondria was detected under different conditions. At least 100 cells were counted per condition (Scale bar = 20 μm) (*n* = 3). **C** The levels of ROS, ATP and OCR were measured to evaluate mitochondrial function in H/R-treated cardiomyocytes, which was transfected with ZFP36L2 shRNA or shRNA NC. JC-1staining (2 µg/ml) was used to analyze mitochondrial membrane potential (△Ψm) (Scale bar = 50 μm) (*n* = 3). **D** Expression of mitochondrial fusion-related genes Drp1, Fis1, Mff, Mfn1, and Mfn2 after cardiomyocytes H/R treatment was evaluated by western blot (*n* = 3). **E** Representative micrographs from immunofluorescence assays of cardiomyocytes stained for Drp1 (green) and the nucleus (DAPI) (Scale bar = 100 μm) (*n* = 3). **P < 0.01; ***P < 0.001.

To further extend our findings in vitro, we used a H/R model with cardiomyocytes treated with 1% O_2_ (hypoxia) for 4 h followed by reoxygenation 1 h. As previously observed, ZFP36L2 levels were increased upon H/R injury (Fig. 2A). Mitochondrial fission, as observed by staining of mitochondria with MitoTracker Red, revealed a 50% increase in fission upon H/R induction. This effect was significantly reduced in the ZFP36L2 knockdown group when compared to the non-targeting control-treated group (Fig. 2B). Furthermore, to understand whether ZFP36L2 was related to mitochondrial function, mitochondrial membrane potential (△Ψm), mitochondrial OCR, ROS, and adenosine triphosphate (ATP) production were measured. These results indicated that mitochondrial ROS was increased under H/R treatment and ZFP36L2 knockdown could significantly alleviate this effect. On the contrary, ATP production, OCR and mitochondrial membrane potential were lower under H/R treatment compared to controls and this reduction was lost upon ZFP36L2 knockdown (Fig. 2C). Similarly, qRT-PCR, western blot and immunofluorescence assays showed that proteins mediating fission or fusion were significantly decreased or increased, respectively, upon knockdown of ZFP36L2 (Supplementary Fig. S2 and Fig. 2D, E). Together, these results showed that in vitro regulation of ZFP36L2 levels alter mitochondrial function processes in response to H/R treatment.

### ZFP36L2 binds to lncRNA PVT1 and regulates its expression

**Fig. 3:**
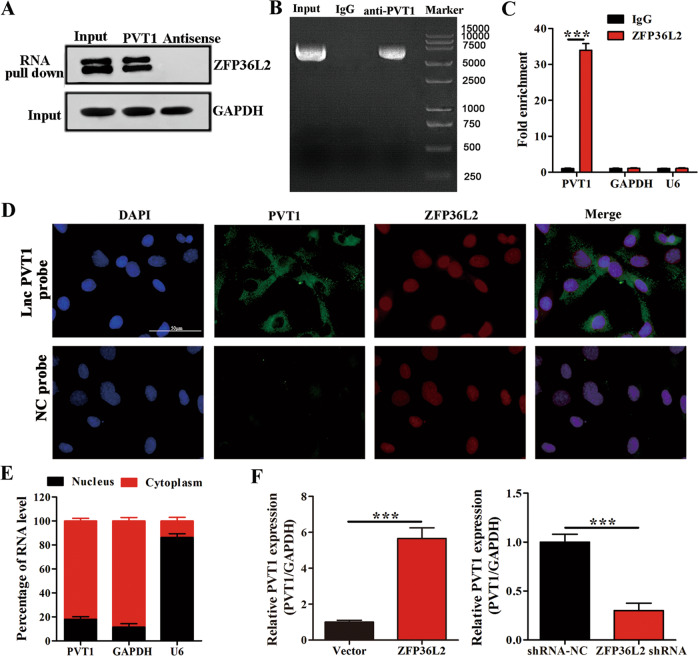
ZFP36L2 binds to lncRNA PVT1 and regulates PVT1 expression. **A** Western blot analyses of association between ZFP36L2 and lncRNA PVT1 using RNA pull-down assay using PVT1 or antisense RNA. GAPDH was used as a loading control (*n* = 3). **B**, **C** Representative northern blots from RIP experiments performed using ZFP36L2 or IgG, indicating the expression of PVT1. Quantification of fold enrichment of PVT1, GAPDH and U6 in ZFP36L2 RIP fractions relative to input RNA is shown (*n* = 3). **D** Representative confocal micrographs from fluorescence in situ hybridization with probes against PVT1 (green) and ZFP36L2 (red). The nucleus was stained with DAPI (Scale bar = 50 μm) (*n* = 3). **E** Total percentage of PVT1 RNA in nuclear and cytoplasmic fractions in cardiomyocytes determined by subcellular fractionation (*n* = 3). **F** PVT1 mRNA levels were detected by qRT-PCR in cardiomyocytes expressing shRNA-NC/ZFP36L2 shRNA and Vector/ZFP36L2 (*n* = 3). ^***^P < 0.001.

To identify the mechanism underlying ZFP36L2-mediated regulation of I/R injury, we performed RNA pull-down and immunoprecipitation experiments. The lncRNA PVT1 was bound to ZFP36L2 and was significantly enriched in the pull-down fraction (Fig. 3A–C). DNA FISH and subcellular fractionation assays showed that a fraction of PVT1 was localized in the cytoplasm, while ZFP36L2 was localized in the nucleus (Fig. 3D, E). We also observed that knockdown of ZFP36L2 reduced the expression of PVT1 and overexpression increased PVT1 levels (Fig. 3F). However, knockdown and overexpression of PVT1 did not affect ZFP36L2 levels (Supplementary Fig. S3). These results indicate that ZFP36L2 binds to PVT1 and directly regulates its expression.

### PVT1 knockdown suppresses I/R injury and sustains mitochondrial function in vivo and in vitro

**Fig. 4:**
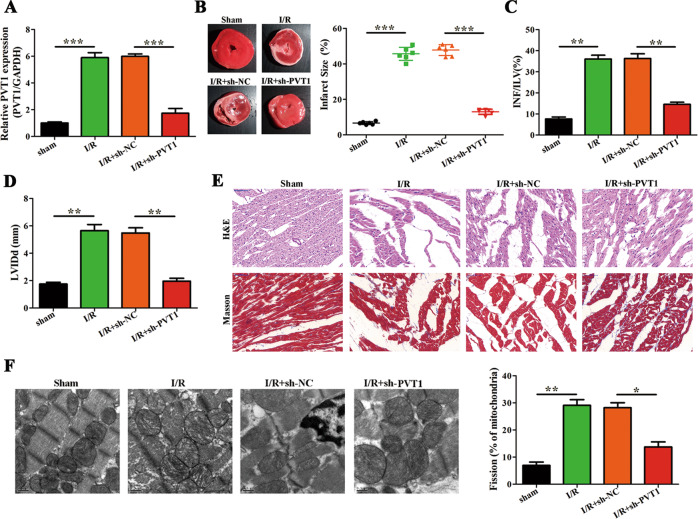
LncRNA PVT1 promotes myocardial I/R injury and mitochondrial fusion and fission in vivo. **A** sh-PVT1 or sh-NC was intraperitoneally administered 24 h before myocardial I/R injury (*n* = 6 per group). Post I/R injury, RNA was isolated from heart tissues and expression of PVT1 was determined by qRT-PCR. Relative fold change of PVT1 expression with respect to GAPDH is shown (*n* = 6). **B**–**D** Quantification of I/R injury. Representative images of heart tissues in the left panel for different groups are shown. Other panels indicate infarct size (%), INF/LV (%), and LVIDd (mm) (*n* = 6). **E** Representative images of H&E and Masson’s trichrome staining of left ventricle [43] sections (Scale bar = 50 μm) (*n* = 6). **F** Representative TEM images of heart tissues are shown in the left panel. Quantification of fragmented mitochondria is shown in the right panel (Scale bar = 0.5 μm) (*n* = 6). *P < 0.05; **P < 0.01; ***P < 0.001.

To verify the functional implication of the association between PVT1 and ZFP36L2, we used an in vivo I/R injury model to determine the effects of PVT1. Upon intraperitoneal injection of shRNA against PVT1 at 24 h post-I/R injury, we observed a reduction in endogenous levels of PVT1, which increased upon I/R induction (Fig. 4A). This also significantly reduced infarct size, INF/LV ratios and diastolic diameter of the left ventricle (LVIDd) when compared to the sham group (Fig. 4B–D). Tissue analyses showed improved cell architecture and reduced apoptosis with low levels of PVT1 (Fig. 4E). In addition, TEM and qPCR analyses showed decreased levels of mitochondrial fission related proteins: Drp1, Fis1, mff and increased expression of fusion-related proteins: Mfn1, Mfn2 upon knockdown of PVT1 in vivo (Fig. 4F and Supplementary Fig. S4). In in vitro H/R injury models, we also observed that PVT1 expression was higher than the control group that was reduced upon knockdown of PVT1 (Supplementary Fig. S5A). Moreover, analysis of mitochondrial morphology revealed a twofold increase in fission upon H/R induction, and that this effect was significantly reduced upon PVT1 knockdown (Supplementary Fig. S5B). Subsequently, the effect of PVT1 on mitochondrial function was assessed. Mitochondrial ROS was significantly induced under H/R treatment compared to controls and this increase was lost upon PVT1 knockdown. On the contrary, ATP production, OCR and mitochondrial membrane potential (△Ψm) were decreased under H/R treatment compared to controls, but was induced upon PVT1 knockdown (Supplementary Fig. S5C). Furthermore, western blot, qRT-PCR analyses and immunofluorescence assays showed increased levels of mitochondrial fusion and fission upon knockdown of PVT1 under H/R treatment (Supplementary Fig. S6A–C). Together, these results indicate that PVT1 knockdown suppresses I/R injury by maintaining mitochondrial function in vivo and in vitro.

### PVT1 directly binds to miR-21-5p and suppresses its activity

**Fig. 5:**
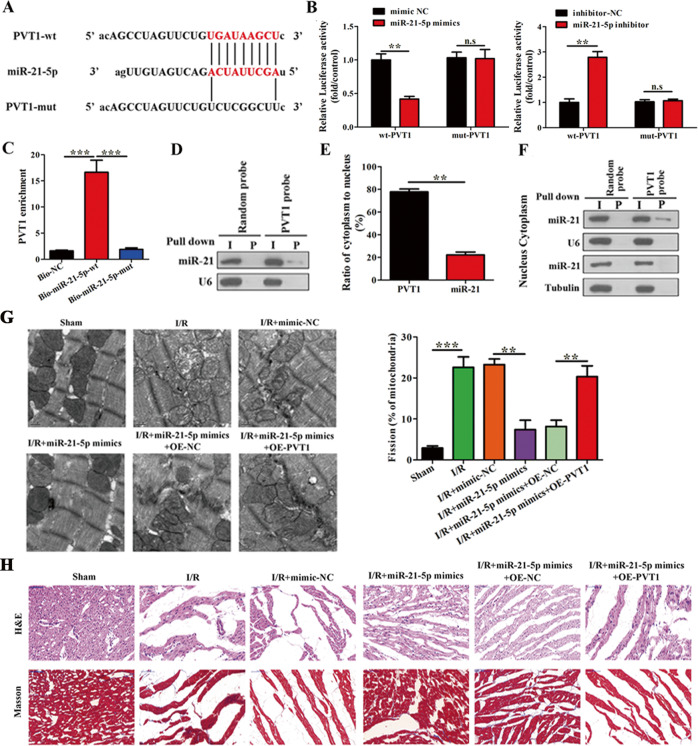
PVT1 directly binds to miR-21-5p and suppresses its activity. **A** Predicted binding sites of PVT1 and miR-21-5p using Starbase 3.0. **B** The luciferase reporter assay was performed in cardiomyocytes co-transfected with mimic-NC, miR-21-5p mimic, or inhibitor-NC, miR-21-5p inhibitor, and PVT1-wt or PVT1-mut (*n* = 3). **C** Cardiomyocytes were transfected with biotinylated WT miR-21-5p (Bio-miR-21-5p-wt) or biotinylated mutant miR-21-5p (Bio-miR-21-5p-mut). A biotinylated miRNA that was not complementary to PVT1 was used as the normal control (Bio-NC). 48 h post transfection, the cells were harvested for a biotin-based pull-down assay and PVT1 expression levels were analyzed by qRT-PCR (*n* = 3). **D** Representative northern blot evaluating expression of miR-21-5p in random or in PVT1 DNA probe pull-down fractions (*n* = 3). **E**, **F** Detection of nuclear or cytoplasmic fractions of PVT1 and miR-21-5p in cardiomyocytes was determined by northern blotting. Tubulin and proliferating cell nuclear antigen (PCNA) were used as controls for cytoplasmic and nuclear fractions, respectively (*n* = 3). **G** Representative TEM images of heart tissues under different conditions of miR-21-5p mimic treatment and PVT1 overexpression (OE-PVT1). Quantification of fragmented mitochondria is shown in the right panel (Scale bar = 0.5 μm) (*n* = 6). **H** Representative images of H&E and Masson’s trichrome staining of LV sections (Scale bar = 50 μm) (*n* = 3). ^**^P < 0.01; ^***^P < 0.001.

PVT1 is known to regulate functions of miRNAs during tumorigenesis [33, 34]. We identified a region in miR-21-5p having a complementary binding sequence to PVT1, using Starbase 3.0 [35], and also generated a binding-deficient mutant of PVT1 (PVT1-mut) (Fig. 5A). Luciferase assay results showed that treatment with a miR-21-5p mimic significantly reduced luciferase activity, indicating an inhibition in wild-type PVT1 (PVT1-wt) expression. This effect was lost in mut-PVT1 when an inhibitor against miR-21-5p was used (Fig. 5B). A biotin-based pull-down assay with miR-21-5p and a mutant version that did not associate with PVT1 showed an enrichment of PVT1 specifically in the miR-21-5p wild-type fraction (Fig. 5C). Additionally, we performed DNA pull-down experiments using PVT1 and observed that miR-21-5p was specifically expressed in the pull-down fraction (Fig. 5D). Fractionation experiments revealed that miR-21-5p specifically associated with PVT1 in the cytoplasm and not in the nucleus (Fig. 5E, F). To test for the effect of miR-21-5p-mediated regulation of PVT1 expression in mitochondrial fission upon I/R injury, we treated I/R induced mice with a miR-21-5p mimic alone or in combination with PVT1 overexpression and/or the respective controls. TEM and tissue analyses revealed that treatment with a miR-21-5p mimic significantly reduced I/R induced effects on mitochondrial fission and cell apoptosis, and that this effect was lost when PVT1 was co-expressed (Fig. 5G, H and Supplementary Fig. S7). In vitro analyses also showed that the total number of cells with fragmented mitochondria was below 20% in the control and miR-21-5p mimic-treated cells, and over 40% when H/R was induced and PVT1 was overexpressed (Supplementary Fig. S8A, B). Drp1 immunofluorescence also showed reduced Drp1 expression in the presence of miR-21-5p mimics under H/R treatment and PVT1 overexpression attenuated this reduction (Supplementary Fig. S8C). Furthermore, we observed that upon knockdown of PVT1, expression of miR-21-5p was significantly higher and overexpression reduced these levels in both in vivo and in vitro analyses (Supplementary Fig. S9A, B). Taken together, these results showed that miR-21-5p and PVT1 are negatively regulated by direct association and alters mitochondrial morphology/function during I/R injury and H/R treatment.

### The miR-21-5p associates with and regulates MARCH5 expression

**Fig. 6:**
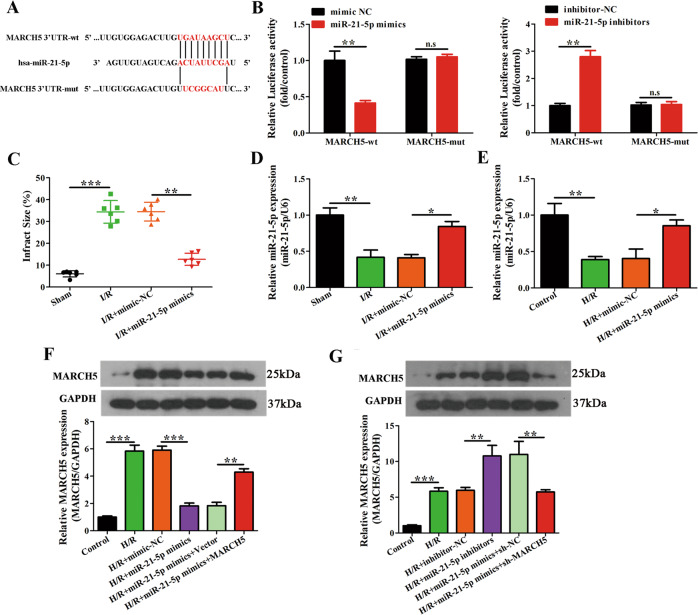
miR-21-5p interactes with MARCH5 and regulates its expression. **A** Binding sites of miR-21-5p and MARCH5 using Starbase 3.0. **B** Relative luciferase activity from reporter assays in cardiomyocytes co-transfected with mimic-NC/miR-21-5p mimic or inhibitor-NC/miR-21-5p inhibitors along with MARCH5-wt or MARCH5-mut (*n* = 3). **C**, **D** The mimic-NC and miR-21-5p mimic were intraperitoneally administered 24 h before myocardial I/R injury and infarction size (%) and levels of miR-21-5p expression were evaluated (*n* = 6). **E** Cardiomyocytes were transfected with mimic-NC and miR-21-5p mimic, followed by induction of H/R. Expression of miR-21-5p was evaluated by qRT-PCR (*n* = 3). **F**, **G** Cardiomyocytes were co-transfected with mimic-NC/miR-21-5p mimic and Vector/MARCH5 or inhibitor-NC/miR-21-5p inhibitor and sh-NC/sh-MARCH5 followed by induction of H/R. MARCH5 expression was evaluated by western blot and qRT-PCR (*n* = 3). *P < 0.05; **P < 0.01; ***P < 0.001 and n.s no significance.

To further understand how miR-21-5p regulates PVT1 mediated mitochondrial fission, we looked for known components binding to miR-21-5p using Starbase [35], and identified MARCH5, an E3 ubiquitin ligase that promotes mitochondrial fission by Drp1 [36, 37]. MARCH5 has a complementary sequence to miR-21-5p in its 3′-UTR region (Fig. 6A). We verified binding by treating cells encoding luciferase under the promoter of wild-type MARCH5 (MARCH5-wt) or a mutant version that could not associate with miR-21-5p (MARCH5-mut). Luciferase assays in cardiomyocytes showed reduced expression of MARCH5-wt upon treatment with a miR-21-5p mimic, and this effect was lost in the MARCH5-mut. Luciferase expression was increased when an inhibitor against miR-21-5p was used (Fig. 6B). Moreover, compared with I/R injury group, miR-21-5p mimics could significantly reduce the infract size in vivo (Fig. 6C). Both in vitro and in vivo, cardiomyocytes responded to treatment with the miR-21-5p mimic by showing increased mRNA levels of miR-21-5p when compared to treatment with the NC mimic (Fig. 6D, E). Treatment with the miR-21-5p mimic also reduced MARCH5 mRNA and protein levels in cardiomyocytes under H/R treatment, the addition of MARCH5 overexpression could reverse this effect; and miR-21-5p inhibitor increased MARCH5 mRNA and protein levels in cardiomyocytes under H/R treatment, this promotion was suppressed by the addition of MARCH5 knockdown (Fig. 6F, G). Together, these results identified MARCH5 to be negatively regulated by miR-21-5p in cardiomyocytes.

### MARCH5 promotes mitochondrial fission in vivo and in vitro

**Fig. 7:**
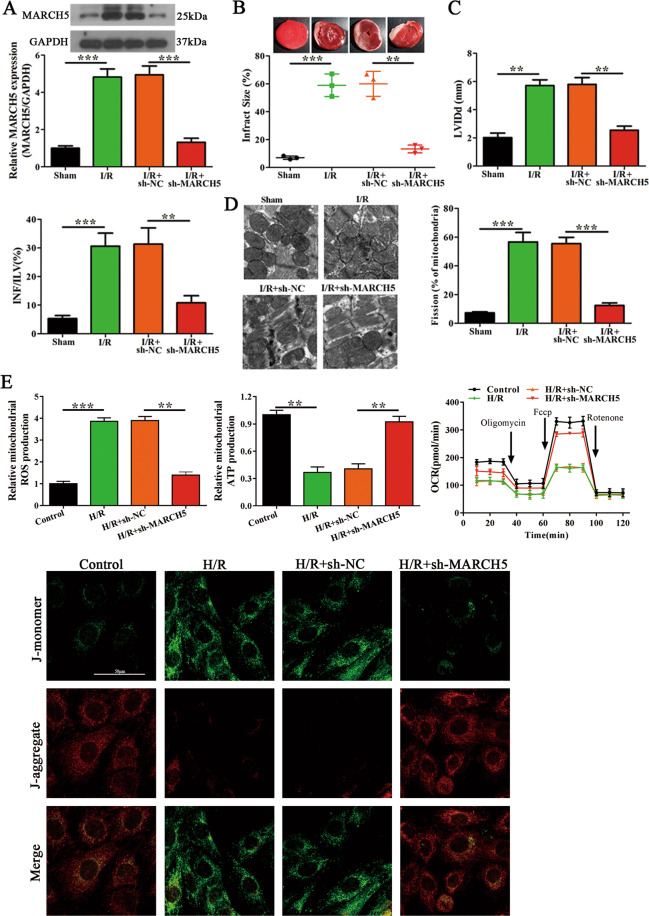
MARCH5 promotes myocardial ischemia/reperfusion (I/R) injury and regulates mitochondrial fusion and fission in vivo. **A** The sh-MARCH5 or sh-NC was intraperitoneally administered 24 h before myocardial I/R injury (*n* = 6). Post-I/R injury, RNA was isolated and the expression of MARCH5 was determined by qRT-PCR and western blot. **B**, **C** The left panel shows representative images of heart tissues. The right panels show the infarct size (%), infarct area/left ventricle (INF/LV) and LVIDd (*n* = 6). **D** Representative TEM images of heart tissues and quantification of fragmented mitochondria (%) (Scale bar = 0.5 μm) (*n* = 6). **E** The levels of ROS, ATP and OCR were measured to evaluate mitochondrial function in H/R-treated cardiomyocytes, which were transfected with sh-MARCH5 or sh-NC. JC-1staining (2ug/ml) was used to analyze mitochondrial membrane potential (△Ψm) (*n* = 3). **P < 0.01; ***P < 0.001.

We next tested the in vivo and in vitro relevance of MARCH5 expression in I/R or H/R injury. Intraperitoneal treatment of shRNA MARCH-treated cardiomyocytes 24 h post-I/R injury significantly reduced MARCH5 expression compared to shNC-treated mice. Similar results were found under H/R treatment in vitro compared to controls (Fig. 7A and Supplementary Fig. S10A). Infarct size decreased more than 50% in the shMARCH5-treated group compared to the controls (Fig. 7B). A significant decrease in LVIDd and INF/LV % was also observed upon shMARCH5 treatment (Fig. 7C). Ultrastructural analyses of TEM images revealed reduced mitochondrial fission, and histology also showed improved tissue architecture and reduced apoptosis in the shMARCH5-treated group (Fig. 7D and Supplementary Fig. S11A). Knockdown of MARCH5 significantly reduced Drp1 mRNA expression along with Fis1 and Mff. However, Mfn1 and Mfn2 levels were increased under I/R injury (Supplementary Fig. S11B). In vitro analyses also showed decreased fragmentation in mitochondria upon treatment with shMARCH5, and reduced Drp1, Fis1, and Mff mRNA and protein expression along with increased Mfn1, Mfn2 mRNA, and protein levels (Supplementary Fig. S10B–E). Moreover, the effect of MARCH5 knockdown on mitochondrial function experiments was also made. Mitochondrial ROS was significantly increased under H/R treatment compared to controls, but this promotion was inhibited by MARCH5 knockdown. On the contrary, the ATP production, OCR and mitochondrial membrane potential (△Ψm) were lower under H/R treatment compared to controls, this reduction was restored by MARCH5 knockdown (Fig. 7E). These results indicate that MARCH5 is functionally implicated in mitochondrial dysfunction during I/R and H/R injury.

### PVT1 regulates mitochondrial fusion and fission through the miR-21-5p/MARCH5 axis in cardiomyocytes

**Fig. 8:**
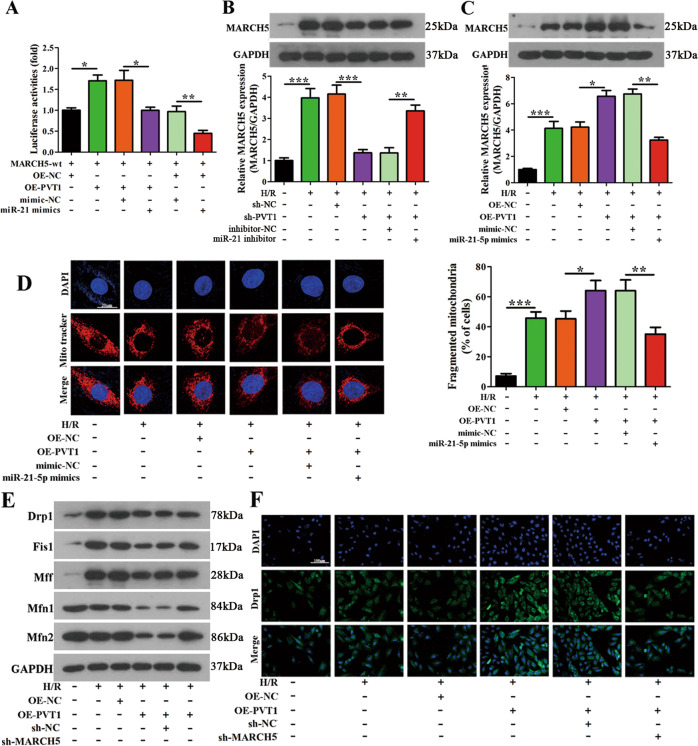
PVT1 regulates mitochondrial fusion and fission through the miR-21-5p/MARCH5 axis. **A** Relative luciferase activity analyzed in 293T cells co-transfected with OE-PVT1 or OE-NC, miR-21-5p mimic, or mimic-NC along with luciferase reporter vector MARCH5 3′-UTR-wt (*n* = 3). **B**, **C** Cardiomyocytes were co-transfected with inhibitor-NC/miR-21-5p inhibitor and sh-NC/sh-MARCH5 or OE-NC/OE-PVT1 and mimic-NC/miR-21-5p mimics followed by exposure to H/R. MARCH5 expression was evaluated by qRT-PCR and western blot (*n* = 3). **D** Mitochondrial fission induced by H/R was determined in cardiomyocytes co-transfected with OE-NC/OE-PVT1 and mimic-NC/miR-21-5p mimics. The % cells with fragmented mitochondria were counted (Scale bar = 20 μm). At least 100 cells were counted per condition (*n* = 3). **E** Expression of mitochondrial fusion-related genes Drp1, Fis1, Mff, Mfn1, and Mfn2 after H/R injury was evaluated by western blot (*n* = 3). **F** Representative confocal micrographs of immunofluorescence staining for Drp1 in cardiomyocytes co-transfected with OE-NC/OE-PVT1 and sh-NC/sh-MARCH5 during H/R injury. The nucleus was stained with DAPI (Scale bar = 100 μm) (*n* = 3). ^*^P < 0.01; ^**^P < 0.01; ^***^P < 0.001.

To assess the roles of lncRNA PVT1, miR-21-5p, and MARCH5 in I/R injury, we tested different conditions in vitro using miR-21-5p mimics, PVT1 overexpression, and shRNA-mediated MARCH5 knockdown. Luciferase expression in response to MARCH5 3′-UTR in 293T cells was increased upon PVT1 overexpression, and this effect was reduced when treated with a miR-21-5p mimic (Fig. 8A). In vitro, cardiomyocytes showed decreased MARCH5 expression when PVT1 was targeted by shRNA (sh-PVT1) and these levels were increased when treated with a miR-21-5p inhibitor, whereas overexpression of PVT1 and treatment with miR-21-5p showed an opposite effect (Fig. 8B, C). Overexpression of PVT1 also increased mitochondrial fragmentation and mitochondrial fission as indicated by Drp1, Fis1, Mff expression levels, and decreased mitochondrial fusion indicated by Mfn1 and Mfn2 expression levels. However, this effect was lost upon co-expression of shMARCH5 as Drp1, Fis1, Mff mRNA, and proteins levels were reduced and Mfn1 and Mfn2 mRNA and protein levels were increased under H/R treatment (Fig. 8D–F and Supplementary Fig. S12). Taken together, these results showed that MARCH5 plays a key role in the interplay between miR-21-5p-mediated regulation of PVT1 expression and its effects on mitochondrial morphology during H/R injury.

## Discussion

Mitochondria are enriched in cardiomyocytes and undergo constant fission and fusion in response to physiological conditions. I/R causes changes in structure and function that determine various cell functions [38, 39]. In this study, we showed that lncRNA PVT1 plays a critical role in altering mitochondrial fission and fusion processes. The levels of PVT1 were regulated by the presence of the zinc finger protein, ZFP36L2, which directly associated with PVT1. Furthermore, we also showed that miR-21-5p negatively regulates PVT1 and MARCH5 levels, thereby reducing mitochondrial fission during I/R injury.

Mitochondrial division during mitoptosis involves pro-apoptotic BAX and BAK proteins along with Drp1, in contrast to the roles of Fis1 and Mdv1 (mitochondrial division 1) proteins during the fission processes [40]. High levels of Drp1 result in morphologically altered mitochondria and reduction in total numbers per cell, along with increased cytochrome c levels and cell death [41]. We observed that Drp1 levels along with Mff and Fis1 were increased upon induction of I/R or H/R. These levels were decreased upon knockdown of ZFP36L2 or PVT1. LncRNAs are classical regulators of gene expression. ZFP36L2 is a nuclear-DNA encoded lncRNA that acts in the mitochondria. Transport of these lncRNAs by RNA binding proteins during I/R injury remains to be elucidated. LncRNA can also form complex three-dimensional RNA-RNA hybrid structures to which zinc finger proteins such as PVT1 can bind and regulate their trans-modulatory effects [42]. Future studies directed at identifying the binding motif of ZFP36L2, which associates with lncRNA PVT1, may provide the possibility to regulate its effects in mitochondrial fission/fusion processes.

In other cell types such as skeletal muscles, PVT1 is localized to both nuclear and cytoplasmic fractions [11], whereas in cardiomyocytes it was largely expressed in the cytoplasm. We found that miR-21-5p preferentially associated with PVT1 in the cytoplasm and reduced its expression. This effect was rescued by treatment with a miR-21-5p inhibitor. Although it is known that PVT1 regulates functions of miRNA to promote cell proliferation and invasion in cancer [43, 44], it is still not clear how I/R injury, and interference with mitochondrial fission/fusion processes are directed by PVT1 and miR-21-5p. MiR-21-5p could bind to fission proteins such as Fis1 or Mff to inhibit its translation as shown previously for miR-484 and miR-761 in cardiomyocytes and specific to I/R injury respectively [45, 46]. Indirectly, it could inhibit Drp1-mediated pathway thereby suppressing p53 mediated triggering of apoptosis.

MiRNAs are capable of interacting with the 3′-UTR regions of mRNAs to alter posttranscriptional modifications or translation [47], and several miRNAs, such as miR-27 and miR-30 have been shown to alter the mitochondrial fission/fusion process [48, 49]. We identified MARCH5 as a possible binding factor to miR-21-5p, and previous studies have shown that MARCH5 alters Drp1-mediated mitochondrial fission by redirecting Drp1 from fission sites [37]. We showed that miR-21-5p bound to the 3′-UTR region of MARCH5, and its knockdown reduced mitochondrial fission in I/R injury. Knockdown of MARCH5 may prevent ubiquitination and subsequent proteasomal degradation of miR-21-5p, thereby retaining fusion properties of mitochondria [50]. We observed that in the presence of MARCH5 and overexpression of PVT1, mitochondrial fission was significantly induced, and these effects were lost upon MARCH5 knockdown. While our data suggests that regulation of mitophagy by MARCH5 is via targeting miR-21-5p, MARCH5 is also capable of directly ubiquitinating fission/ fusion proteins [26, 27]. Therefore there is a possibility of additional mechanisms and direct targets for MARCH5 to coordinate mitochondrial morphogenesis during I/R injury in cardiomyocytes.

Hence, we have described a novel mechanism of miR-21-5p/MARCH5-mediated regulation of mitochondrial morphology by PVT1 during I/R injury. Among increasing data describing a role for miRNAs as a link between I/R injury and mitochondrial dysfunction, our study contributes towards further understanding these mechanisms, which may eventually translate into improved therapeutic approaches.

## Supplementary information

Supplementary Figure legend

Supplementary Figure S1

Supplementary Figure S2

Supplementary Figure S3

Supplementary Figure S4

Supplementary Figure S5

Supplementary Figure S6

Supplementary Figure S7

Supplementary Figure S8

Supplementary Figure S9

Supplementary Figure S10

Supplementary Figure S11

Supplementary Figure S12

Supplementary Table S1

## Data Availability

The datasets used and/or analyzed during the current study are available from the corresponding author on reasonable request.
