# Supplementary material for: ZFP36L2 regulates myocardial ischemia/reperfusion injury and attenuates mitochondrial fusion and fission by LncRNA PVT1
Source: Cell Death Dis. 2021 Jun 15;12(6):614. doi: 10.1038/s41419-021-03876-5 (PMC8206151; doi:10.1038/s41419-021-03876-5)
Supplement: Supplementary file 1 — Supplementary Figure legend [file 41419_2021_3876_MOESM1_ESM.docx]

**Fig.S1 (A)** ZFP36L2 and Vector were intraperitoneally administered 24 h before induction of I/R injury. The upper panel shows representative images of tissue samples. The below panel indicates infarction size (%) (n=6). (**B**) ZFP36L2 shRNA and shRNA-NC were intraperitoneally administered 24 h before induction of I/R injury. Expression of mitochondrial fission and fusion-related genes Drp1, Fis1, Mff, Mfn1 and Mfn2 after myocardial I/R injury was evaluated by qRT-PCR (n=6). *P < 0.05, **P < 0.01 and ***P < 0.001.

**Fig.S2** A H/R injury model was established by inducing hypoxia for 4 h followed by reoxygenation for 1 h in cardiomyocytes, which was transfected with ZFP36L2 shRNA or shRNA-NC. Expression of mitochondrial fission and fusion-related genes Drp1, Fis1, Mff, Mfn1 and Mfn2 after H/R treatment in cardiomyocytes was evaluated by qRT-PCR (n=3). ***P* < 0.01 and ****P* < 0.001.

**Fig.S3** Cardiomyocytes were transfected with sh-NC/ sh-PVT1 or Vector/PVT1. The mRNA levels of ZFP36L2 in cardiomyocytes were detected by qRT-PCR (n=3). n.s= no significance.

**Fig.S4** sh-PVT1 or sh-NC was intraperitoneally administered 24 h before induction of I/R injury. Expression of mitochondrial fission and fusion-related genes Drp1, Fis1, Mff, Mfn1, and Mfn2 after myocardial I/R injury was evaluated by qRT-PCR (n=6). **P* < 0.05 and ***P* < 0.01.

**Fig.S5 LncRNA PVT1 facilitates cardiomyocytes H/R injury and affectes mitochondrial function *in vitro*.** (**A**) Expression of PVT1 after H/R treatment was detected by qRT-PCR (n=3). (**B**) Cardiomyocytes were transfected with sh-PVT1 and sh-NC followed by induction of H/R and stained using Mito Tracker Red and DAPI (Scale bar=20μm) (n=3). The % cells with fragmented mitochondria were counted (n=3). (**C**) The levels of ROS, ATP and OCR were measured to evaluate mitochondrial function in H/R-treated cardiomyocytes, which were transfected with sh-PVT1 or sh-NC. JC-1staining (2ug/ml) was used to analyze mitochondrial membrane potential (△Ψm). (Scale bar=50μm) (n = 3). ^*^*P* < 0.05; ^**^*P* < 0.01 and ^***^*P* < 0.001.

**Fig.S6 PVT1 knockdown could increase mitochondrial fusion and fission *in vitro*.** Cardiomyocytes were transfected with sh-PVT1 and sh-NC followed by H/R treatment. (**A-B**) Expression of mitochondrial fusion-related genes *Drp1*, *Fis1*, *Mff*, *Mfn1*, and *Mfn2* after H/R injury was evaluated by qRT-PCR and western blot (n=3). (**C**) Representative confocal micrographs of immunofluorescence staining for Drp1 in cardiomyocytes upon H/R injury. The nucleus was stained with DAPI (Scale bar=100μm) (n=3). **P* < 0.05 and ***P* < 0.01.

**Fig.S7** mimic-NC/miR-21-5p mimics and OE-NC/OE-PVT1 were intraperitoneally administered 24 h before induction of I/R injury. Expression of mitochondrial fusion-related genes *Drp1*, *Fis1*, *Mff*, *Mfn1*, and *Mfn2* after I/R injury was evaluated by qRT-PCR (n=6). ^*^*P* < 0.05; ^**^*P* < 0.01 and ^***^*P* < 0.001.

**Fig.S8 PVT1 modulates mitochondrial fission and fusion via miR-21-5p.** Cardiomyocytes were co-transfected with mimic-NC/miR-21-5p mimics and OE-NC/OE-PVT1 followed by H/R treatment. **(A-B)** Expression of mitochondrial fusion-related genes *Drp1*, *Fis1*, *Mff*, *Mfn1*, and *Mfn2* after H/R injury was evaluated by qRT-PCR and western blot (n=3). (**C**) Immunofluorescence staining for Drp1 was determined after H/R treatment. The nucleus was stained with DAPI (Scale bar=100μm) (n=3). ^*^*P* < 0.05 and ^**^*P* < 0.01.

**Fig.S9** (**A**) sh-PVT1/sh-NC or OE-PVT1/OE-NC was intraperitoneally administered 24 h before induction of I/R injury. Relative miR-21-5p expression was evaluated by qRT-PCR (n=6). (**B**) Relative miR-21-5p expression was evaluated by qRT-PCR in cardiomyocytes, which were co-transfected with sh-PVT1/sh-NC and OE-PVT1/OE-NC followed by induction of H/R (n = 3). *P < 0.05; **P < 0.01 and ***P < 0.001.

**Fig.S10 MARCH5 increases cardiomyocytes H/R and modulates mitochondrial fusion and fission *in vitro*.** (A) Expression of MARCH5 was detected by qRT-PCR under H/R treatment (n=3). (**B-C**) Cardiomyocytes were transfected with sh-NC or sh-MARCH5 followed by H/R treatment. Expressions of mitochondrial fission and fusion-related genes *Drp1*, *Fis1*, *Mff*, *Mfn1*, and *Mfn2* after H/R injury was evaluated by qRT-PCR and western blot (n=3). (**D**) Immunofluorescence staining for Drp1 determined after H/R treatment. The nucleus was stained with DAPI (Scale bar=100μm). (**E**) Representative micrographs of cardiomyocytes transfected with sh-MARCH5 or sh-NC followed by induction of H/R and stained with MitoTracker Red and DAPI (Scale bar=20μm) (n=3). Cells with fragmented mitochondria were quantified. At least 100 cells were counted per condition (n = 3). ^**^*P* < 0.01 and ^***^*P* < 0.001.

**Fig.S11** sh-MARCH5 and sh-NC were intraperitoneally administered 24 h before induction of I/R injury. (**A**) Representative images of H&E. Masson’s trichrome and IHC staining or immunodetection of MARCH5 expression in LV sections (Scale bar=50μm) (n=6). (**B**) Expression of mitochondrial fission/fusion-related genes Drp1, Fis1, Mff, Mfn1 and Mfn2 was evaluated by qRT-PCR (n = 6). **P < 0.01 and ***P < 0.001.

**Fig.S12** Cardiomyocytes were co-transfected with OE-NC/OE-PVT1 and sh-NC/sh-MARCH5 followed by H/R treatment. Expression of mitochondrial fission/fusion-related genes Drp1, Fis1, Mff, Mfn1 and Mfn2 under H/R treatment was evaluated by qRT-PCR (n = 3). **P* < 0.05; ***P* < 0.01 and ****P* < 0.001.
