# Supplementary figures and images for: ZFP36L2 regulates myocardial ischemia/reperfusion injury and attenuates mitochondrial fusion and fission by LncRNA PVT1
Source: Cell Death Dis. 2021 Jun 15;12(6):614. doi: 10.1038/s41419-021-03876-5 (PMC8206151; doi:10.1038/s41419-021-03876-5)

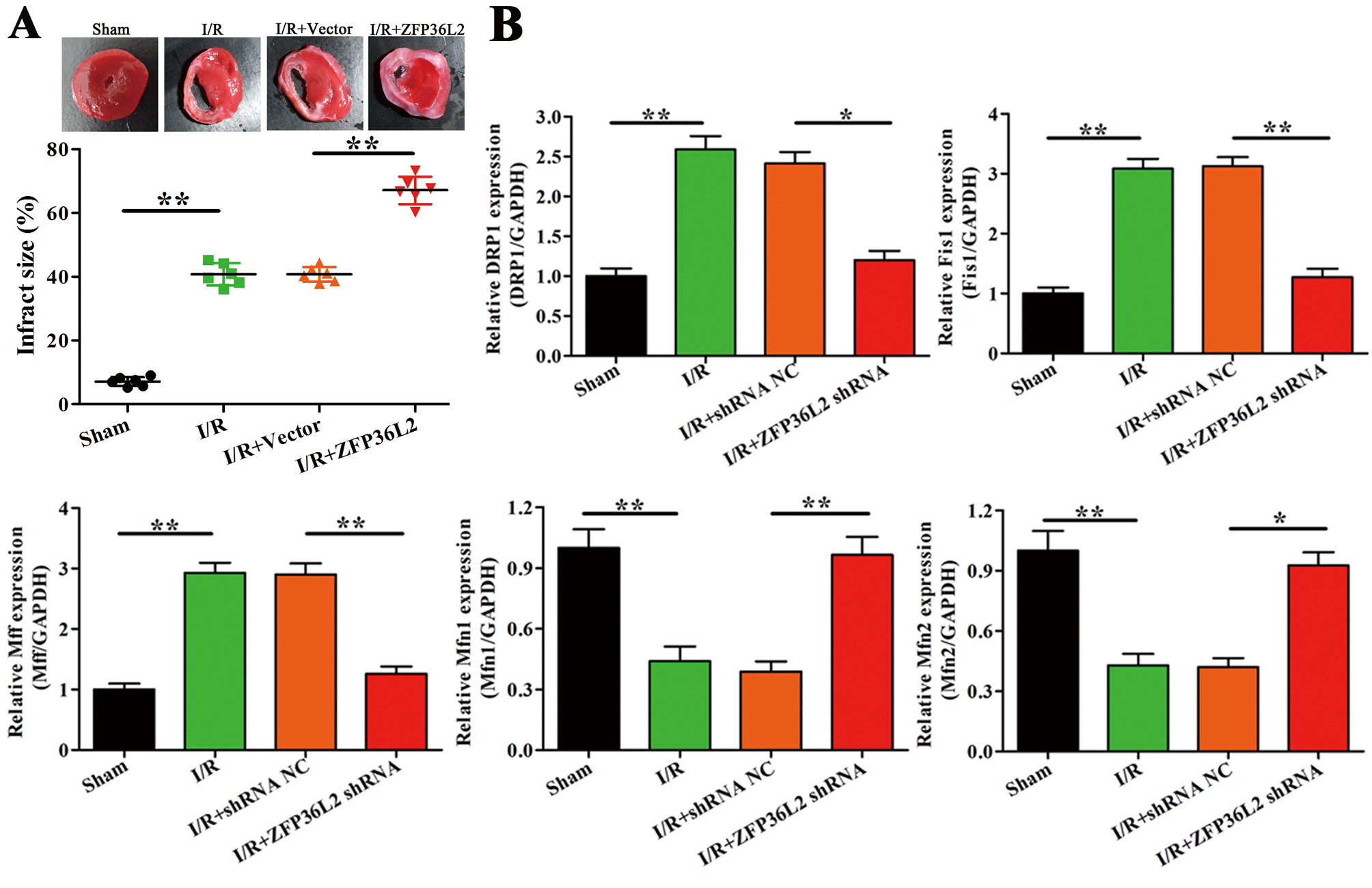

Supplement: Supplementary file 2 — Supplementary Figure S1 [file 41419_2021_3876_MOESM2_ESM.tif]

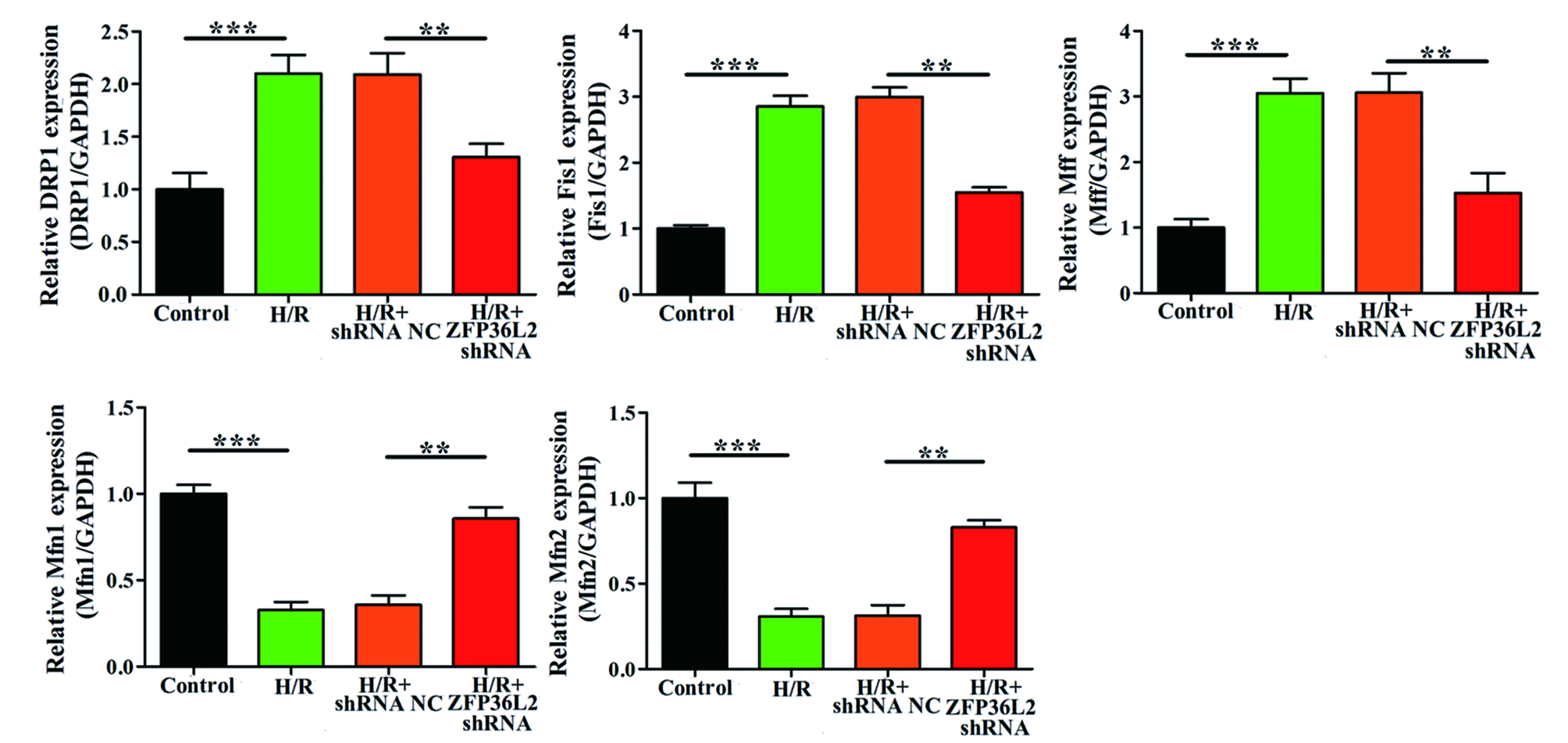

Supplement: Supplementary file 3 — Supplementary Figure S2 [file 41419_2021_3876_MOESM3_ESM.tif]

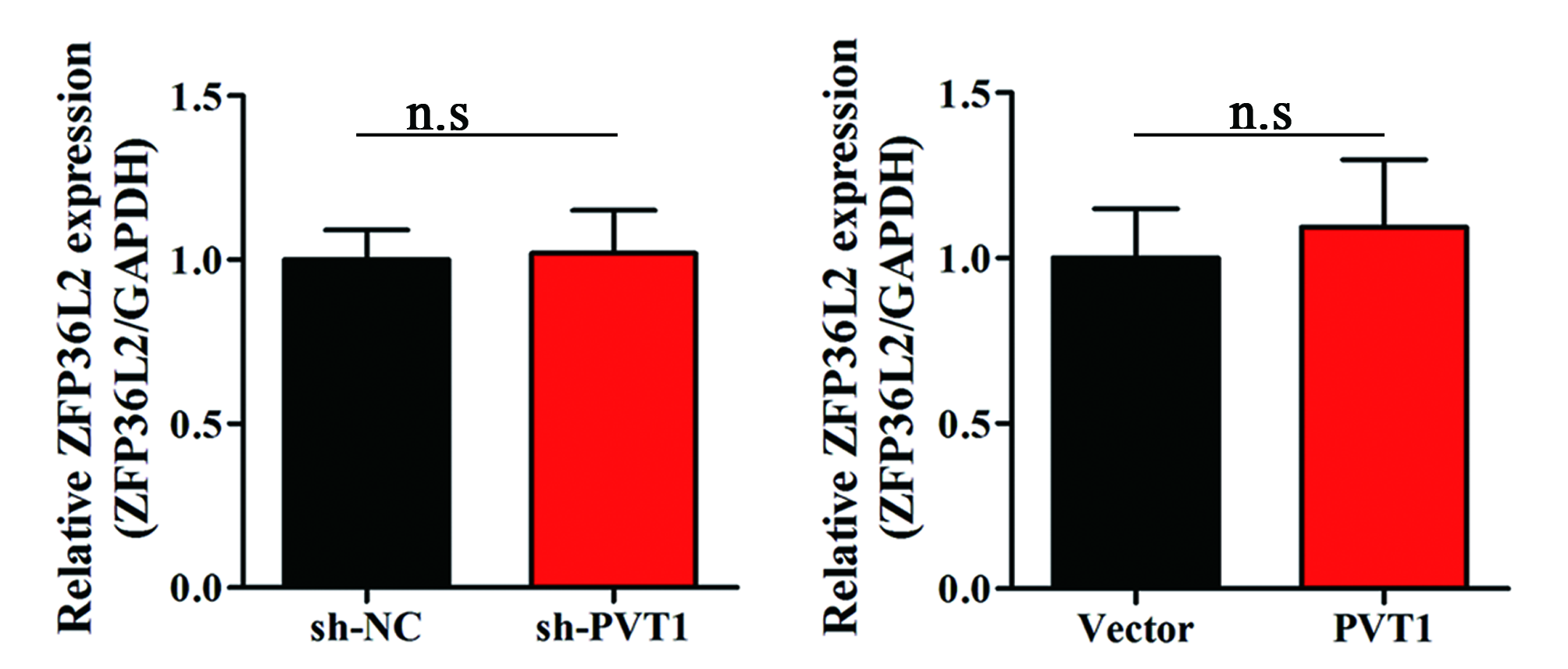

Supplement: Supplementary file 4 — Supplementary Figure S3 [file 41419_2021_3876_MOESM4_ESM.tif]

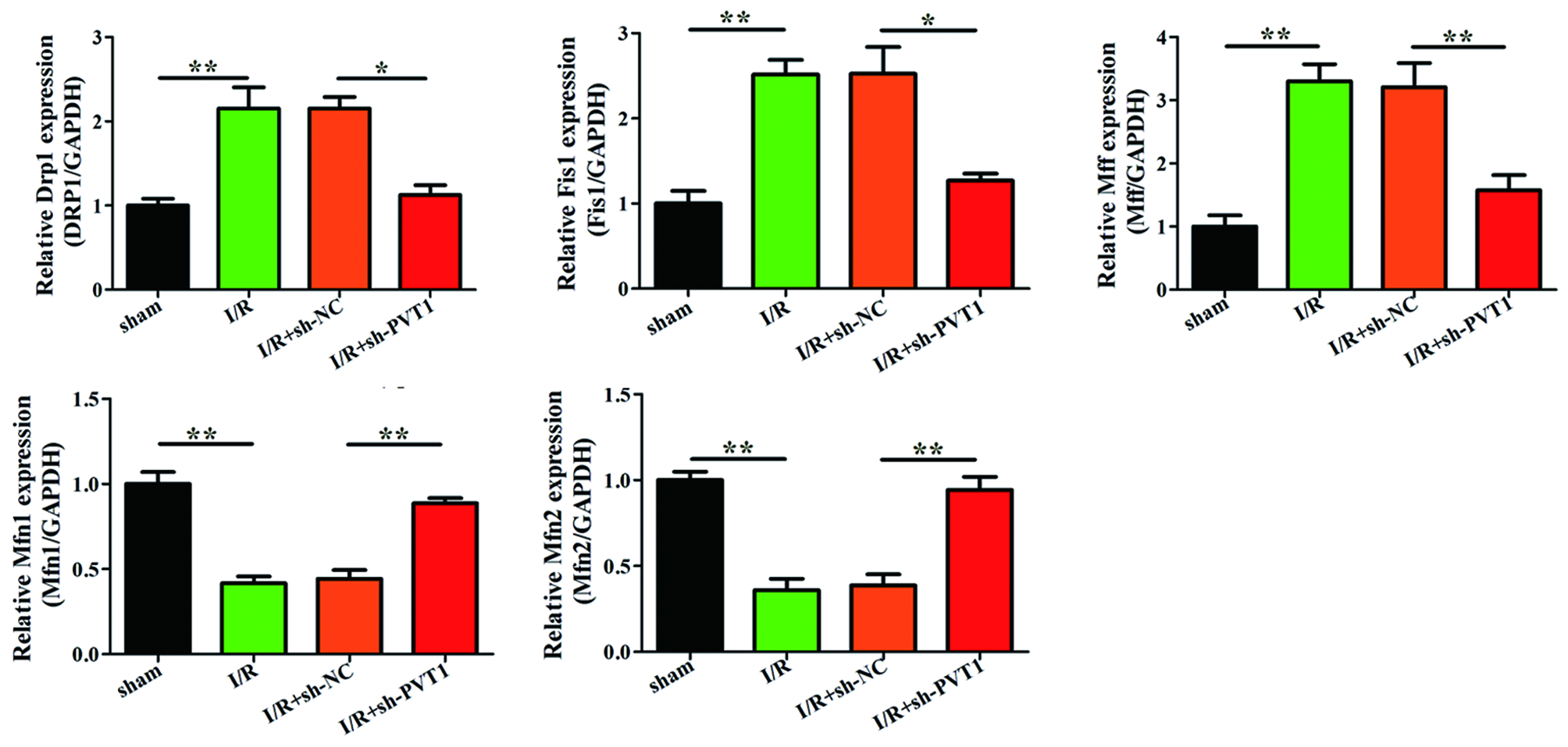

Supplement: Supplementary file 5 — Supplementary Figure S4 [file 41419_2021_3876_MOESM5_ESM.tif]

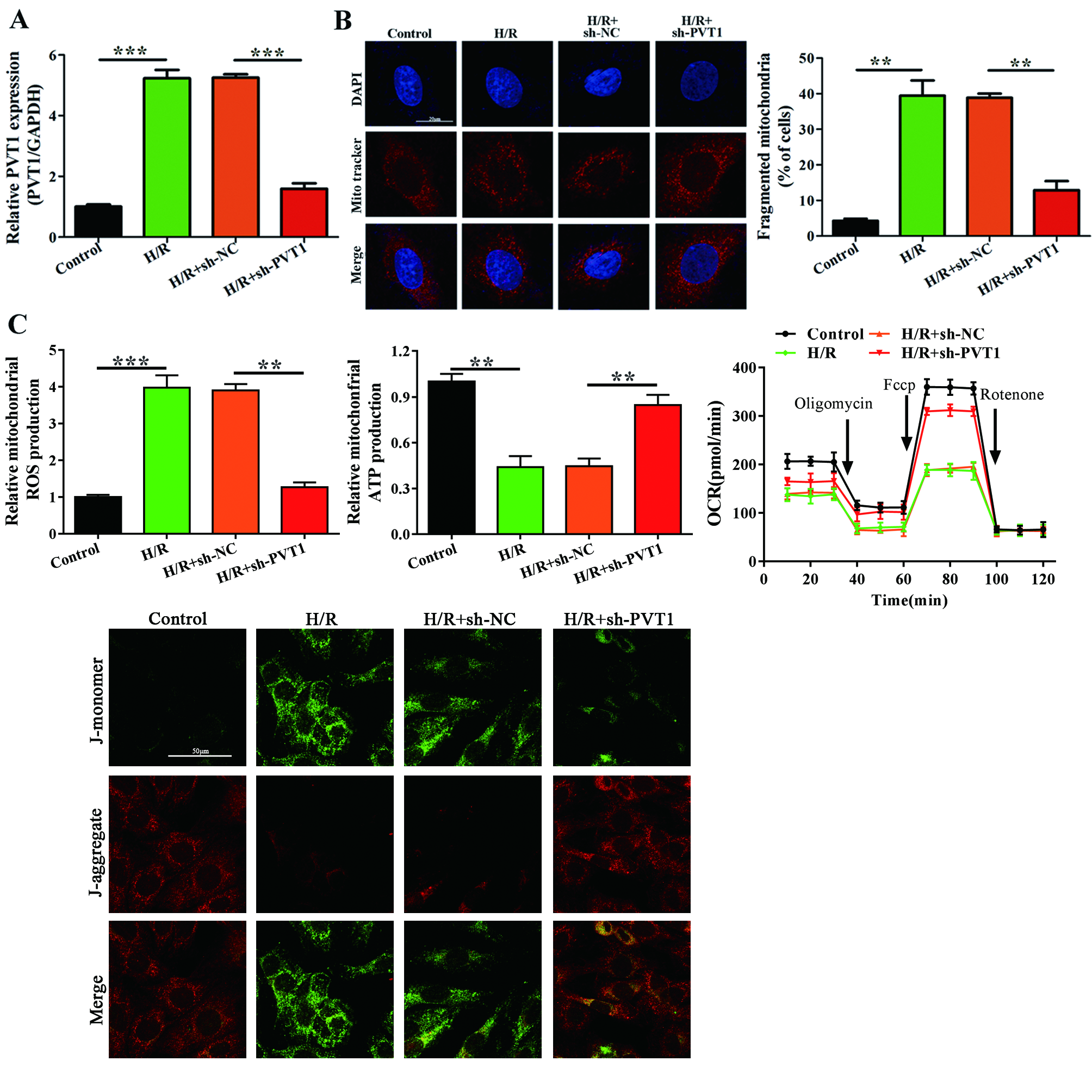

Supplement: Supplementary file 6 — Supplementary Figure S5 [file 41419_2021_3876_MOESM6_ESM.tif]

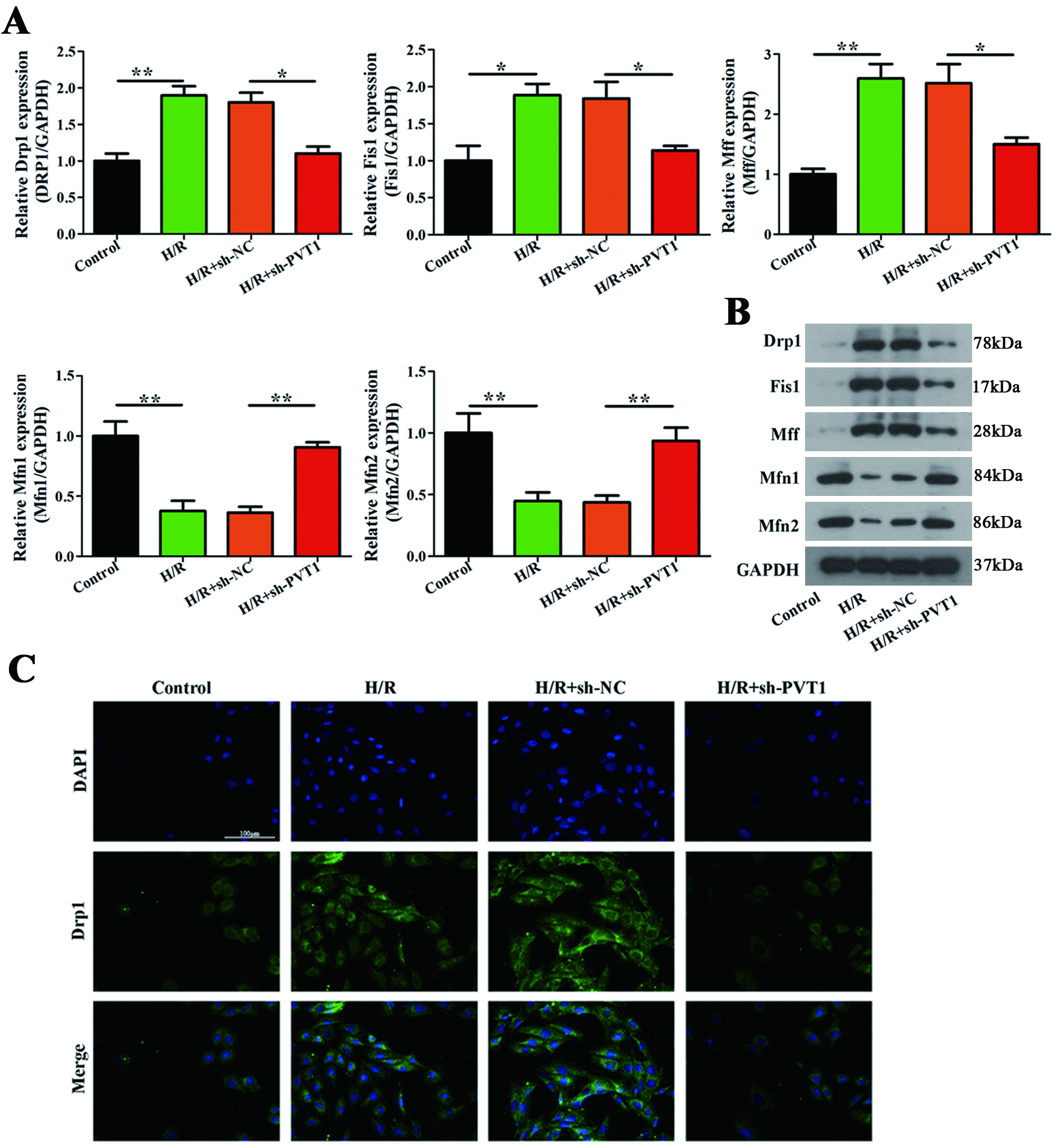

Supplement: Supplementary file 7 — Supplementary Figure S6 [file 41419_2021_3876_MOESM7_ESM.tif]

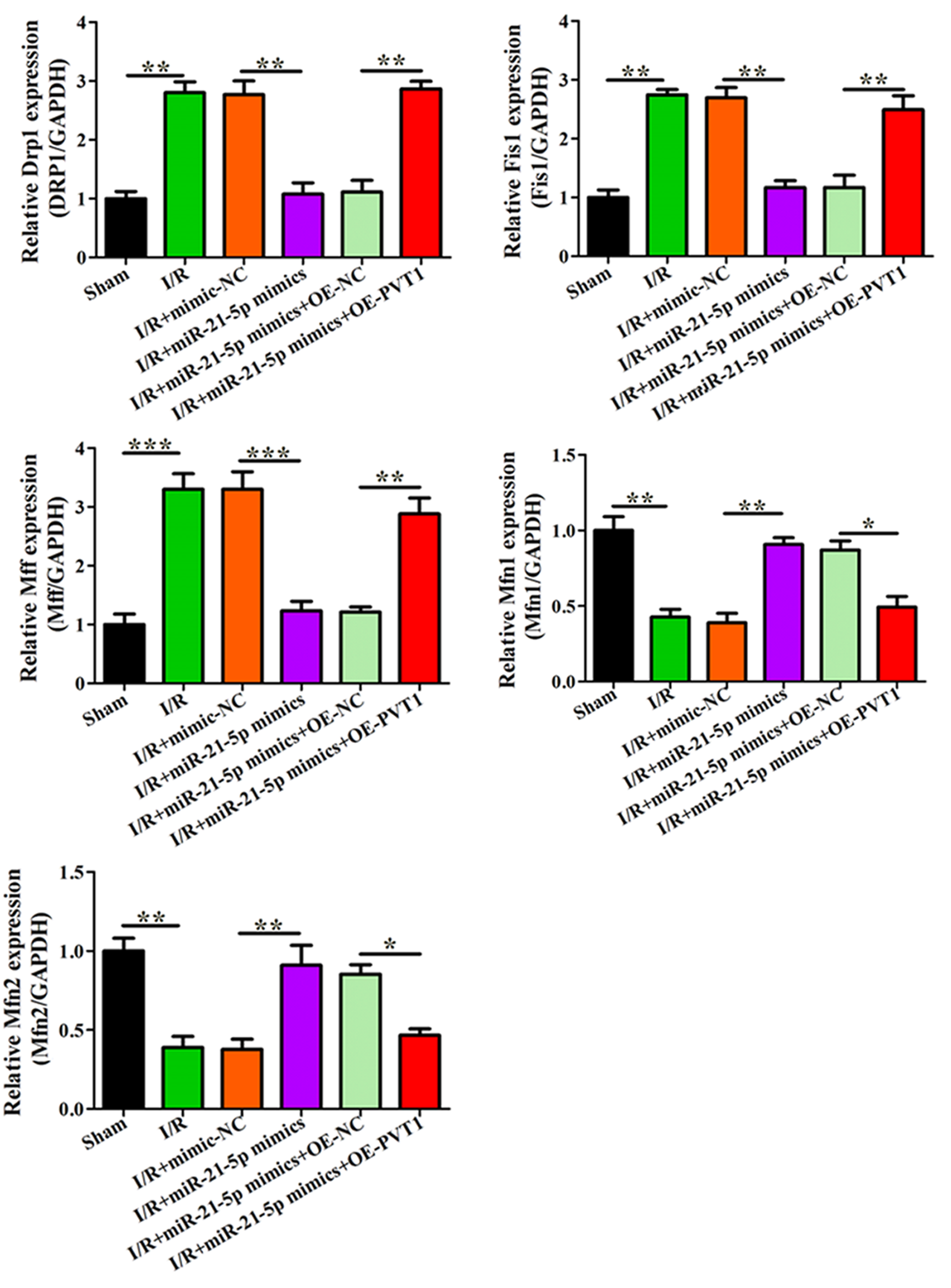

Supplement: Supplementary file 8 — Supplementary Figure S7 [file 41419_2021_3876_MOESM8_ESM.tif]

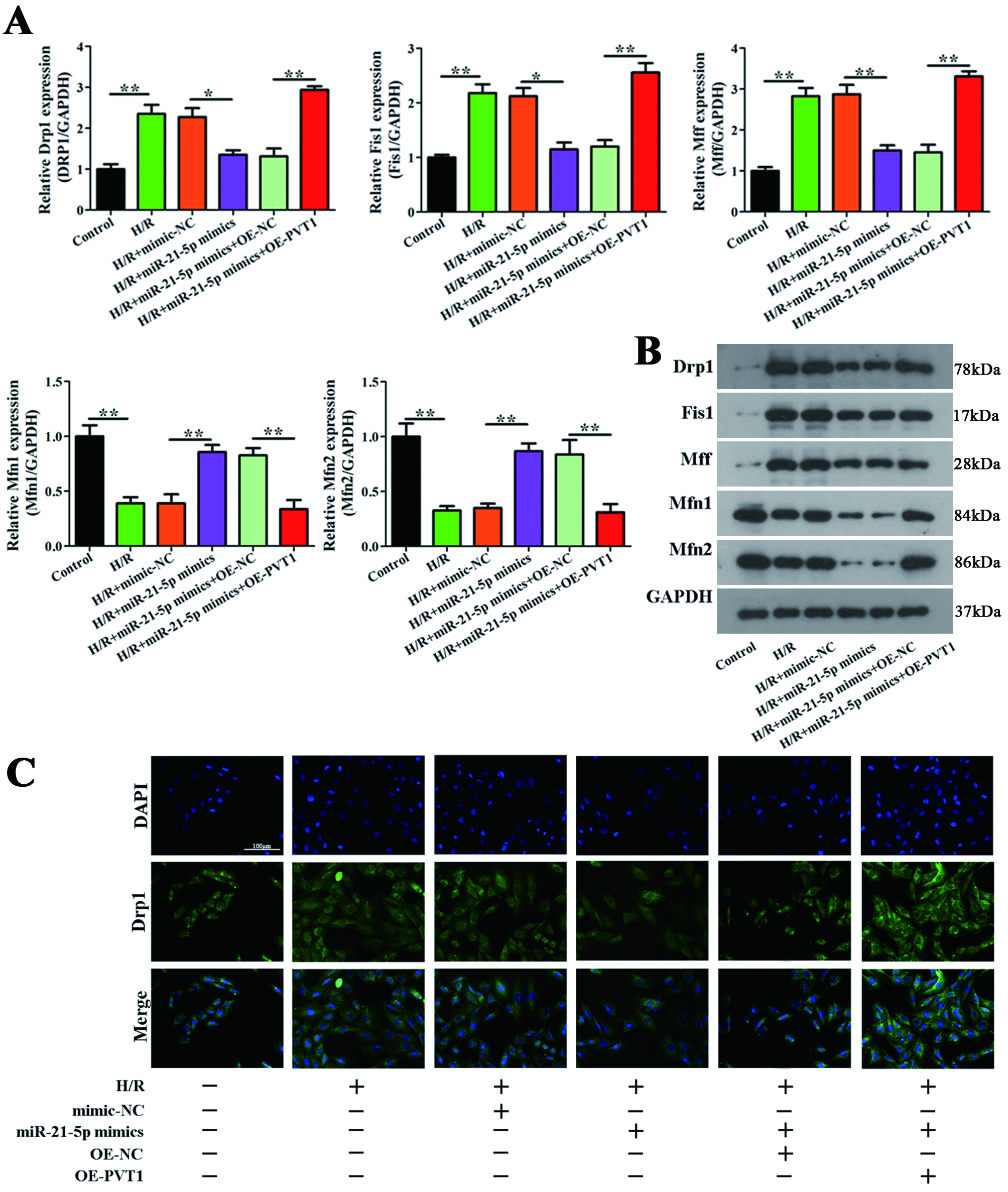

Supplement: Supplementary file 9 — Supplementary Figure S8 [file 41419_2021_3876_MOESM9_ESM.tif]

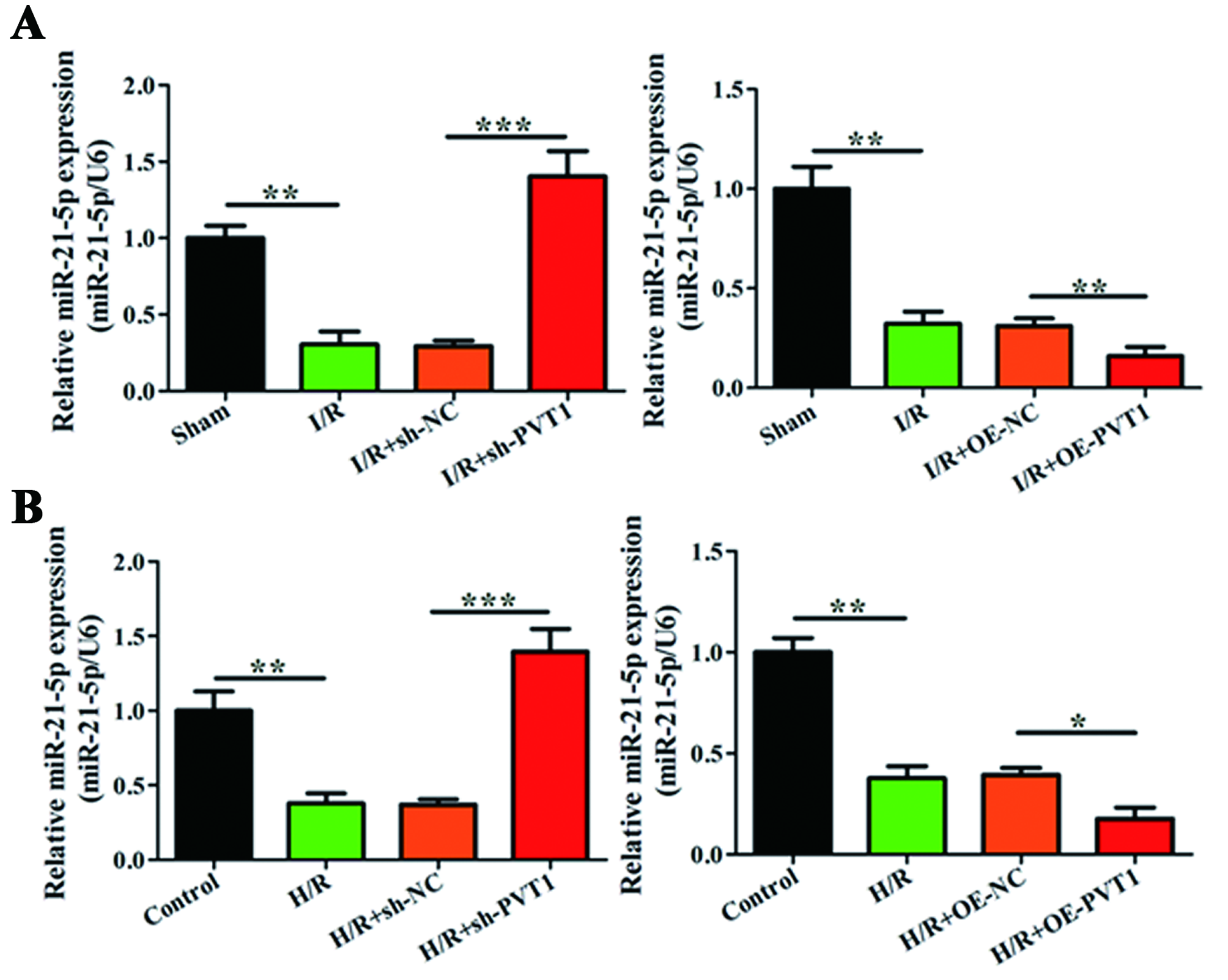

Supplement: Supplementary file 10 — Supplementary Figure S9 [file 41419_2021_3876_MOESM10_ESM.tif]

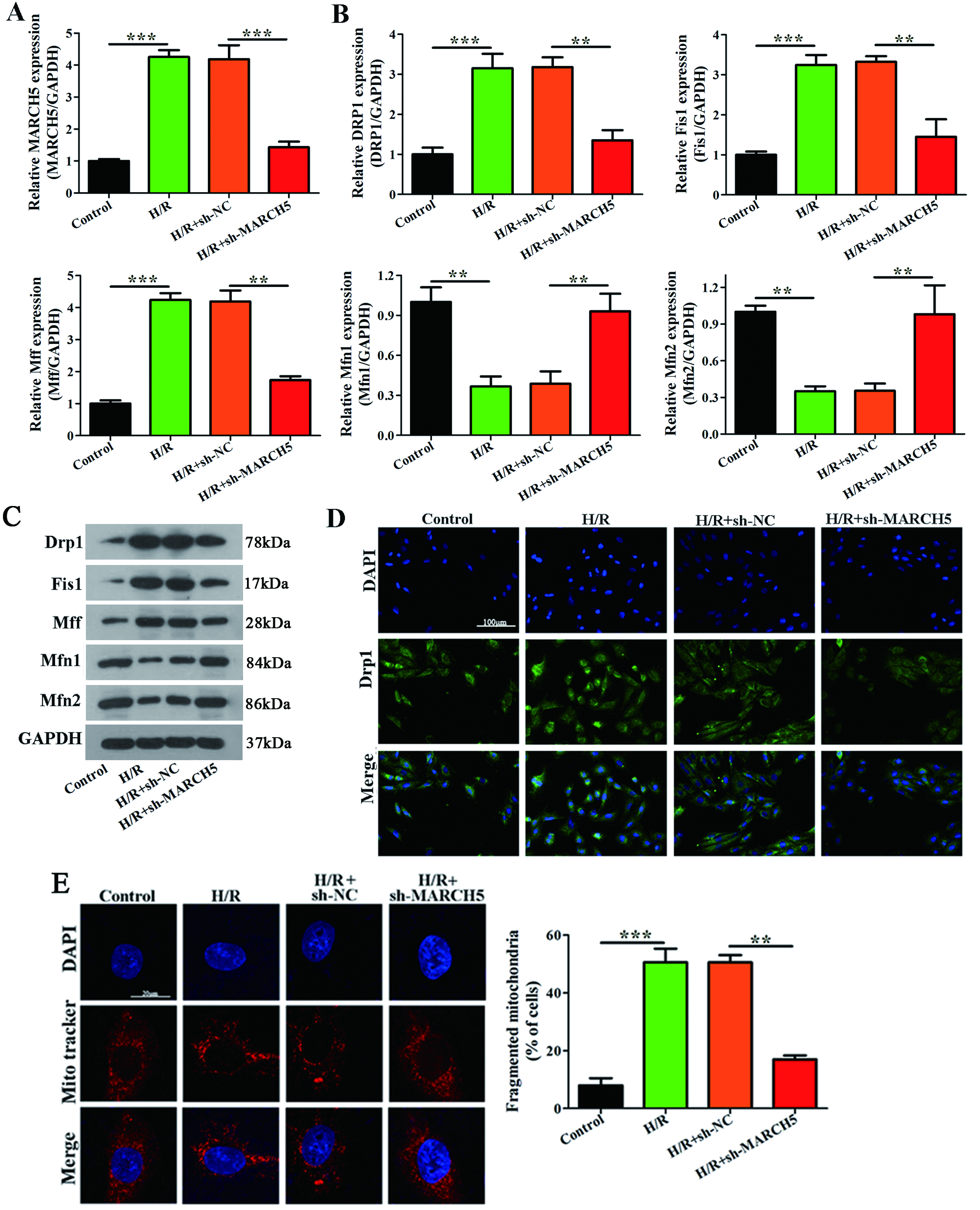

Supplement: Supplementary file 11 — Supplementary Figure S10 [file 41419_2021_3876_MOESM11_ESM.tif]

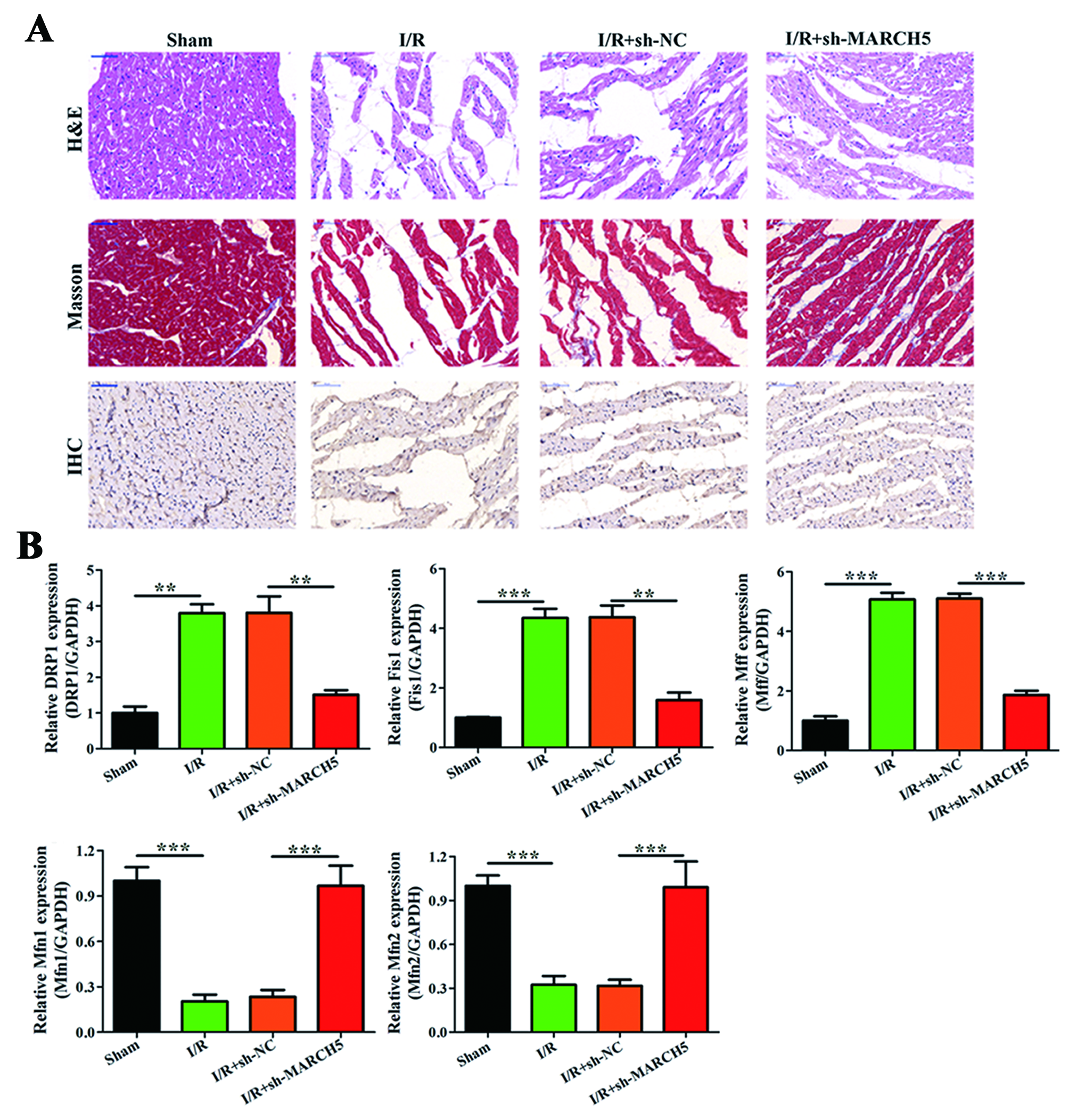

Supplement: Supplementary file 12 — Supplementary Figure S11 [file 41419_2021_3876_MOESM12_ESM.tif]

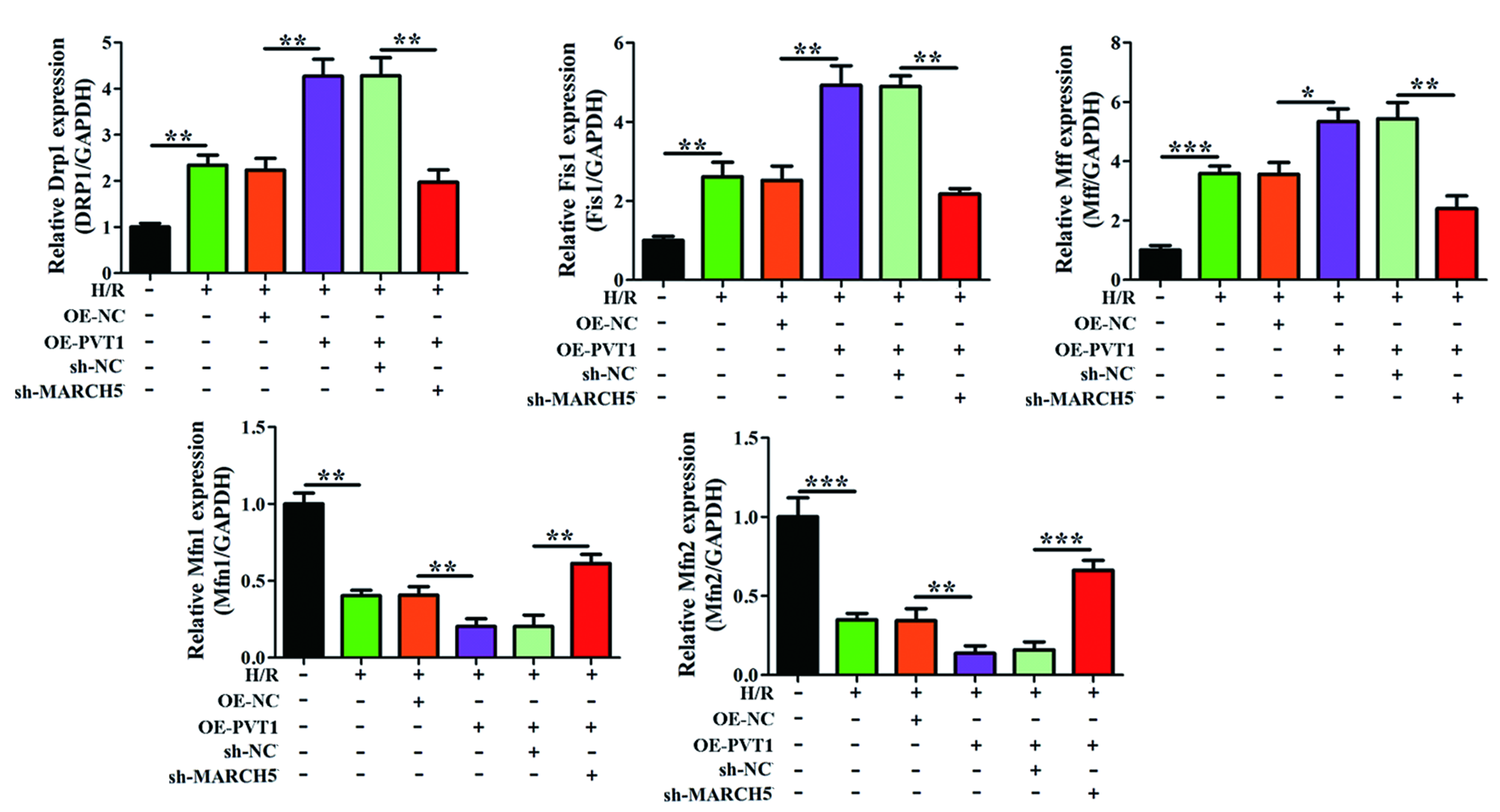

Supplement: Supplementary file 13 — Supplementary Figure S12 [file 41419_2021_3876_MOESM13_ESM.tif]
