# Supplementary material for: ZFP36L2 regulates myocardial ischemia/reperfusion injury and attenuates mitochondrial fusion and fission by LncRNA PVT1
Source: Cell Death Dis. 2021 Jun 15;12(6):614. doi: 10.1038/s41419-021-03876-5 (PMC8206151; doi:10.1038/s41419-021-03876-5)
Supplement: Supplementary file 14 — Supplementary Table S1 [file 41419_2021_3876_MOESM14_ESM.docx]

| **Table S1. The sequences for the primers** | | |
| --- | --- | --- |
| Gene | Primer direction | Sequence |
| ZFP36L2 | Forward | 5'-ATCAACTCCACGCGCTACAA-3' |
|  | Reverse | 5'-GGCAGAAGCCGATGGTATGA-3' |
| PVT1 | Forward | 5'-TGAGAACTGTCCTTACGTGACC-3' |
|  | Reverse | 5'-AGAGCACCAAGACTGGCTCT-3' |
| MiR-21-5p | Forward | 5'- CGCGCTAGCTTATCAGACTGA -3' |
|  | Reverse | 5'- CGGCCCAGTGTTCAGACTAC -3' |
| U6 | Forward | 5'-CTCGCTTCGGCAGCACA-3' |
|  | Reverse | 5'-AACGCTTCACGAATTTGCGT-3' |
| MARCH5 | Forward | 5'-CCAAAATTGGGTCCAGTGGTTT-3' |
|  | Reverse | 5'-GACAAGGGCTCCACACAAGA-3' |
| DRP1 | Forward | 5'-AGAAAATGGGGTGGAAGCAGA-3' |
|  | Reverse | 5'-CAGGCACCTTGGTCATTCCT-3' |
| Fis1 | Forward | 5'- TGTCCAAGAGCACGCAGTTT -3' |
|  | Reverse | 5'- GAGCAGCACGATGCCTTTAC -3' |
| Mff | Forward | 5'-CGTGCTCTCAGCCAACCA -3' |
|  | Reverse | 5'- TGCCAACTGCTCGGATTTCT-3' |
| Mfn1 | Forward | 5'-GAACCACCAAGGAGTGTGGAA-3' |
|  | Reverse | 5'-CCCTCCCATGAAAAGGAAACAT-3' |
| Mfn2 | Forward | 5'-AAAAACGCAATGTCCCTGCT-3' |
|  | Reverse | 5'-CTTCTGTGGTAACGGGGTCC-3' |
| GAPDH | Forward | 5'- TTGGTATCGTGGAAGGACTCA -3' |
|  | Reverse | 5'- TGTCATCATATTTGGCAGGTT -3' |
